# Ether-lipids accumulation promotes hepatocellular carcinoma progression linked to PPARα deficiency

**DOI:** 10.1186/s12929-025-01178-y

**Published:** 2025-09-11

**Authors:** Pei-Yin Liao, Wen-Jen Lin, Pei-Chun Shen, Cian-Ru Yang, Ying-Chun Yu, Chun-Chieh Yeh, Long-Bin Jeng, Hsieh-Chou Lai, Wei-Chung Cheng, Wen-Lung Ma

**Affiliations:** 1 Graduate Institute of Biomedical Sciences, School of Medicine, China Medical University, Taichung, 406040 Taiwan; 2 Department of Medical Research, Organ Transplantation Center, China Medical University Hospital, Taichung, 404327 Taiwan; 3 Ph.D. Program for Health Science and Industry, Center of Tumor Biology, School of Medicine, China Medical University, Taichung, 406040 Taiwan; 4 Department of Gastroenterology and Department of Surgery, China Medical University Hospital, Taichung, 404327 Taiwan; 5 Cancer Biology and Precision Therapeutics Center, Ph.D. Program for Cancer Molecular Biology and Drug Discovery, China Medical University, Taichung, 406040 Taiwan; 6 Office of Research and Development, Asia University, Taichung, 413505 Taiwan; 7 Education Center, National Cheng Kung University Hospital, Tainan, 704302 Taiwan; 8 College of Medicine, National Cheng Kung University, Tainan, 701 Taiwan

**Keywords:** Ether-lipid, PPARα, Prognosis marker, HCC, Lipidomics, Multi-omics

## Abstract

**Background:**

While the Warburg effect links glycolysis to de novo lipid synthesis in carcinogenesis, the roles of lipids in cancer prognosis remain elusive. Here, a multi-omics approach was conducted in a cohort of hepatocellular carcinoma (HCC) to elucidate the role of lipid metabolites as prognostic markers.

**Methods:**

Ninety-eight HCC patients were recruited between 2011 and 2013. Their specimens were subjected to transcriptomic and lipidomic profiling. The resulting data were then analyzed using strategic bioinformatics approaches to identify associations with HCC prognosis. Subsequently, lipid-related pathways implicated in these analyses were verified using cellular and molecular approaches.

**Results:**

Our findings indicate that lipidomic profiling is a potential prognostic marker for HCC. Specifically, higher levels of ether-lipids were significantly associated with poor survival and adverse clinical features, such as advanced TNM stage and metastasis. Analysis of transcriptomic patterns within patient groups defined by lipidomic profiles revealed that ether-lipid abundance inversely correlated with PPAR signaling but positively correlated with the expression of metastasis-associated gene clusters (e.g., genes involved in ECM remodeling, adhesion, and migration). Functional studies verified that ether-lipids enhance cell mobility. Consistent with the proposed mechanism, treatment with a PPARα agonist reduced ether-lipid accumulation and cell mobility. Therefore, we delineated an axis whereby PPARα downregulation leads to ether-lipid accumulation, subsequently promoting cell mobility. Mechanistically, we propose that deficient PPARα-mediated lipophagy results in cellular ether-lipid accumulation. These lipids, in turn, promote cell mobility via Transient Receptor Potential Vanilloid 2 (TRPV2)-mediated cytoskeletal rearrangement.

**conclusion:**

This study identifies lipidome patterns as a risk factor for patient prognosis. Mechanistically, deficient PPARα-mediated lipophagy leads to the accumulation of ether-lipids within cancer cells, which in turn promotes cell mobility via calcium-dependent, TRPV2 channel-mediated cytoskeletal rearrangement.

**Supplementary Information:**

The online version contains supplementary material available at 10.1186/s12929-025-01178-y.

## Introduction

Recent evidence indicates that lipid reprogramming in cancer is not only involved in energy provision and membrane biosynthesis [1] but also an indicator of cancer growth, cell migration, and immune regulation [2]. Specifically, lipids have been identified as energy sources [1, 3] and as components for membrane assembly [1, 4]. Moreover, certain lipids, such as arachidonic acid and eicosanoids, have been implicated in mechanisms for escaping immune surveillance in cancer [5, 6]. These lipids have also been linked to cell survival, for example, through the process of ferroptosis [7]. Regarding cancer progression, lipids have been reported to be metastasis regulators [8]. For instance, the conversion of lysophosphatidylcholine to lysophosphatidic acid (LPA) leads to LPA receptor activation, thereby promoting migratory signals [9]. Furthermore, lipid droplet (LD) accumulation can engender the release of CD36-binding long-chain fatty acids, which facilitates M1/M2 macrophage differentiation and promotes cancer metastasis [10].

Despite successful demonstrations of lipid reprogramming in cancer development, hypothesis-driven approaches have been unable to identify critical lipid metabolites associated with clinical features in a global and unbiased manner. This scenario might be caused by the lack of advanced lipid profiling technologies capable of omics-level and high-throughput analyses [11]. However, with substantial advancements in lipidomics [12], such challenges can be addressed. Several studies have attempted to identify lipid metabolites in various human cancers [13–15] by comparing lipidome expression in normal and tumor tissues; these studies have discovered lipids critical for carcinogenesis. However, no study has conducted lipidome profiling in a cohort to explore the roles of lipids in cancer prognosis.

The origins of tumor lipids might be autonomous or nonautonomous. They may play compensatory or complementary roles during cancer development. For example, the expression of cholesterogenic genes is irrelevant to gastric cancer prognosis [16], whereas the expression of steroidogenic genes is crucial for this prognosis [17]. Cholesterol is a precursor for steroidogenesis, and nonautonomous cholesterol importation through lipoprotein receptors, instead of cholesterogenic genes, promotes favorable prognoses [17]. By contrast, statin (HMG-CoA reductase inhibitor) use is associated with lower cancer risks, as determined by a post-hoc analysis [18]. However, statins have not been shown to prolong survival in prospective trials [19]. These examples highlight two points. First, lipids involved in cancer development are evolving. Second, the relationships of gene expression and lipid expression with cancer prognosis are complex, potentially parallel, and not necessarily causal.

To address the role of lipids in cancer prognosis, we used multiomics measurements in a cohort of consecutive hepatocellular carcinoma (HCC) patients to determine the association of several types of lipids with HCC clinical features. Using a data-oriented approach in an HCC cohort, we can identify important lipid metabolite associations with cancer prognosis. In this report, the abundance and the mechanism of prognostic lipids are explained.

## Materials and methods

### Patient cohort study

We established a cohort of 98 patients with hepatocellular carcinoma (HCC) who underwent hepatectomy at the Organ Transplantation Center of China Medical University Hospital (CMUH), Taiwan, between 2011 and 2013 (Supplementary Figure S1). Tumor specimens were collected, preprocessed, aliquoted, and stored at − 80 °C for subsequent protein, RNA, and lipid extraction; some of the specimens were embedded in paraffin for immunohistochemical (IHC) or histological examination. The cohort’s data were monitored for up to 2000 days. Basic demographic data and survival hazard ratios are presented in Supplementary Figure S1A and S1B. Patient chart data, including follow-up information, were linked to primary HCC tumor specimen data, including lipidomic profiling data and transcriptomic profiling data, for which the patient number of each specimen type is shown in a Venn diagram plot (Supplementary Figure S1C). This study was approved by the Institutional Review Board of CMUH (IRB# DMR100-IRB-088).

### Sample preparation for lipidome and transcriptome analysis

All samples were prepared following the manufacturer’s instructions to perform lipidomic analysis and transcriptome analysis. For lipidome analysis, the liver tissue from the HCC cohort was resuspended in D-PBS (Ca^2+^/Mg^2+^-free) for homogenization. Then, homogenates were prepared at a concentration of 5 mg/ml, and 300 µL aliquots were used for analysis.. Treated cells were resuspended in D-PBS (Ca^2+^/Mg^2+^-free) at a concentration of 3000 cells per μl, and then at least 300 μl of the cell suspension was prepared for analysis. For transcriptome analysis, the HCC cell lines (Tong and Huh7) were passaged three consecutive times. At each passage, 1 × 10^6^ cells were seeded into new culture vessels. After reaching the appropriate confluency, cells were harvested and subjected to RNA isolation. Purified RNA was quantified at OD260 nm using an ND-1000 spectrophotometer (Nanodrop Technology, USA), and RNA quality was assessed using a Bioanalyzer 2100 (Agilent Technologies; add location, e.g., Santa Clara, CA, USA) with an RNA 6000 LabChip kit (Agilent Technologies).

### Lipidome profiling

Lipids were extracted using a two-step chloroform/methanol procedure [20]. Samples were spiked with internal lipid standard mixture containing: cardiolipin 14:0/14:0/14:0/14:0 (CL), ceramide 18:1;2/17:0 (Cer), diacylglycerol 17:0/17:0 (DAG), hexosylceramide 18:1;2/12:0 (HexCer), lyso-phosphatidate 17:0 (LPA), lyso-phosphatidylcholine 12:0 (LPC), lyso-phosphatidylethanolamine 17:1 (LPE), lyso-phosphatidylglycerol 17:1 (LPG), lyso-phosphatidylinositol 17:1 (LPI), lyso-phosphatidylserine 17:1 (LPS), phosphatidate 17:0/17:0 (PA), phosphatidylcholine 17:0/17:0 (PC), phosphatidylethanolamine 17:0/17:0 (PE), phosphatidylglycerol 17:0/17:0 (PG), phosphatidylinositol 16:0/16:0 (PI), phosphatidylserine 17:0/17:0 (PS), cholesterol ester 20:0 (CE), sphingomyelin 18:1;2/12:0;0 (SM) and triacylglycerol 17:0/17:0/17:0 (TAG). After extraction, the organic phase was transferred to an infusion plate and dried in a speed vacuum concentrator. The first-step dry extract was re-suspended in 7.5 mM ammonium acetate in chloroform/methanol/propanol (1:2:4, V:V:V), and 2nd step dry extract in 33% ethanol solution of methylamine in chloroform/methanol (0.003:5:1; V:V:V). All liquid handling steps were performed using the Hamilton Robotics STARlet robotic platform with the Anti-Droplet Control feature for organic solvents pipetting.

Mass spectrometry-based lipidome analysis was performed by Lipotype GmbH (Dresden, Germany). Samples were analyzed by direct infusion on a QExactive mass spectrometer (Thermo Scientific) equipped with a TriVersa NanoMate ion source (Advion Biosciences). Samples were analyzed in both positive and negative ion modes with a resolution of Rm/z = 200 = 280,000 for MS and Rm/z = 200 = 17,500 for MSMS experiments, in a single acquisition. MSMS was triggered by an inclusion list encompassing corresponding MS mass ranges scanned in 1 Da increments. Both MS and MSMS data were combined to monitor CE, DAG, and TAG ions as ammonium adducts; PC, PC O–, as acetate adducts; and CL, PA, PE, PE O–, PG, PI, and PS as deprotonated anions. MS only was used to monitor LPA, LPE, LPE O–, LPI, and LPS as deprotonated anions; Cer, HexCer, SM, LPC, and LPC O– as acetate adducts. Lipid identification was performed using LipidXplorer software. Only lipid identifications with a signal-to-noise ratio > 5, and a signal intensity fivefold higher than in corresponding blank samples, were considered for further data analysis.

### Lipidomics data analysis

We use our previously developed tools [21–23] to perform lipidomics data analysis. In sum, we applied a data filtering process to exclude lipid species with a missing value rate exceeding 70%. For the remaining missing values or values below the limit of detection, we imputed them using half of the minimum detected value for that lipid species across all samples. To address the skewed distribution of lipid abundance measurements, we further performed a log 10 transformation before conducting differential lipid expression analyses.

To visualize the samples by reducing the dimensionality of the processed lipidomics data, the t-SNE (t-Distributed Stochastic Neighbor Embedding) algorithm was used with specific hyperparameters: output dimensionality = 2, perplexity = 15, and iterations = 3000. Subsequently, we applied K-means clustering, an unsupervised learning method, to segregate and label two groups of patients based on the transformed t-SNE data. To identify differentially expressed lipids, we employed two-tailed Student’s t-tests with the Benjamini–Hochberg correction method to calculate fold changes and adjusted p-values between the two groups. Unless otherwise specified, lipid species were considered significant if their adjusted p-values were < 0.05 and their absolute log2 fold change was ≥ 1.

The results of differentially expressed lipid species were utilized for enrichment analysis of various lipid characteristics. Over-representation analysis with Fisher's exact test was applied to identify whether specific categories of lipid characteristics have significantly more or fewer up-regulated or down-regulated lipid species than expected by chance. The cut-off criterion for determining significantly enriched or depleted categories in lipid characteristics was set at a p-value < 0.05.

### Trans-Omics data analysis

We conducted a Spearman correlation analysis to evaluate the associations between lipid abundances and gene expression. We reported the resulting p-values and correlation coefficients to assess the strength of these associations. An association was considered significant if it met the following criteria: an absolute correlation coefficient greater than 0.4 and a p-value less than 0.05. We further analyzed the overlapping genes correlated with ether-lipids using functional enrichment analysis to investigate the biological functions of ether-lipids.

### Survival analysis

We calculated the median value of each lipid species and used it to stratify patients into two groups. Lipidomics data and clinical records, encompassing demographics, liver function parameters, and tumor characteristics, were transformed into binary variables. We constructed a univariate Cox proportional hazards (Cox-PH) model and a log-rank test to investigate survival-related clinical variables, lipid species, and lipid characteristics. This analysis allowed us to calculate log-rank p-values, hazard ratios (HR), and their corresponding 95% confidence intervals. Significant survival-relevant factors were identified based on a log-rank p-value < 0.05. Additionally, for survival analysis, we calculated the median value of each lipid and used it to stratify patients into two groups. Additionally, we conducted enrichment analysis for lipid species in relation to patient prognosis. We employed the hazard ratio (HR) to determine whether specific lipid species were beneficial or detrimental to patient survival.

### Transcriptome profiling

All RNA sample preparation procedures were carried out according to the Illumina's official protocol. Illumina's TruSeq Stranded Total RNA Library Prep Gold Kit (Cat. 20020598) was used for library construction, followed by AMPure XP beads (Beckman Coulter, USA) size selection. The sequence was determined using Illumina's sequencing-by-synthesis (SBS) technology (Illumina, USA) NovaSeq 6000. Sequencing data (FASTQ reads) were generated using Welgene Biotech's pipeline based on Illumina's basecalling program bcl2fastq v2.20. Transcriptomic data are publicly available in Gene Expression Omnibus (GEO) at GSE242315.

### Transcriptomics data analysis

We utilized our previously developed pipelines to analyze the RNA-seq data [24]. In sum, Raw sequencing reads in FASTQ format underwent initial quality assessment using FastQC (v0.11.5) (https://www.bioinformatics.babraham.ac.uk/projects/fastqc/). Subsequently, adapter sequences and low-quality bases were removed using Trimmomatic (v0.39) [25], employing standard parameters for quality trimming and minimum read length filtering. The resulting high-quality, processed reads were aligned to the GRCh38 reference genome from the Ensembl database using the HISAT2 aligner (v2.2.1) [26]. Alignments were processed and sorted before quantification. Transcript assembly and gene-level quantification were performed using StringTie (v2.1.5) [26], guided by the reference genome annotation (Homo_sapiens.GRCh38.97.gtf downloaded from the Ensembl official website). This involved merging transcript information across all samples to create a consensus annotation before calculating final gene expression estimates for each sample. Gene-level read counts derived from the StringTie output were then used for differential expression analysis using the DESeq2 package (v1.30.1) [27] in the R statistical environment (v4.0.0). Genes were considered statistically significant if they met criteria: mean number of transcripts per million was > 1, adjusted p-value < 0.05 (Benjamini–Hochberg correction), and absolute log2 fold change > 1.

Functional enrichment analysis elucidates the biological roles and pathways associated with the identified significantly differentially expressed genes (DEGs). The set of significant DEGs was tested for over-representation in established functional categories, including Gene Ontology (GO) and pathway databases such as KEGG, Wikipathway, and Reactome. P-values were adjusted for multiple comparisons using the Benjamini–Hochberg procedure to control the False Discovery Rate (FDR). Functional categories and pathways with an adjusted p-value below 0.05 were considered significantly enriched.

### Cell culture

Human HCC cell lines (Tong and Huh7) were cultured in Dulbecco’s modified Eagle’s medium (DMEM; Gibco, Carlsbad, CA, USA) supplemented with 10% fetal bovine serum (FBS; Gibco, Carlsbad, CA, USA) and 1% penicillin/streptomycin (30-002-CI; Corning, New York, USA) in a 5% CO2 atmosphere at 37 °C. The Huh7 cell lines were purchased from the Bioresource Collection and Research Center (Hsinchu, Taiwan) and the American Type Culture Collection (Manassas, VA, USA). The Tong cells were generously provided by YS Jou (Academia Sinica, Taiwan).

### Immunoblot and quantitative analyses

For protein extraction from the human liver tissues and HCC cells, samples were lysed in radioimmunoprecipitation assay (RIPA) buffer (100 mM Tris, 5 mM EDTA, 5% NP40; pH 8.0) containing protease inhibitors and kept on ice for 30 min. Lysates were then centrifuged at 14,000 rpm for 25 min to isolate total protein [28]. These proteins were mixed with 6X sample buffer, subjected to sodium dodecyl sulfate–polyacrylamide gel electrophoresis, and subsequently transferred to a polyvinylidene fluoride membrane (Millipore, MA, USA). The membrane was soaked in 5% nonfat milk for 1 h to block nonspecific binding, after which it was incubated with primary antibodies overnight. The following day, the membrane was washed thrice in Tris-buffered saline with Tween 20 (TBST; 1% Triton) for 10 min each and then incubated with secondary antibodies for 1 h. After another three washes in TBST and one in TBS, ECL reagent was applied to the membrane. Protein signals were detected using a Chemidoc XRS + (Bio-Rad) equipped with a charge-coupled device (CCD) camera. All captured images were analyzed using Image J [29].

### RNA isolation and qRT-PCR

All samples from the HCC cells and human liver tissues were lysed with 1 mL TRIzol (15,596,026; Invitrogen, Carlsbad, CA, USA). Phenol–chloroform (pH 6.7/8.0; 0883; VWR International, Pennsylvania, USA) was subsequently added for phase separation, and RNA-rich layers were isolated through centrifugation. Soluble RNA was precipitated using 2-propanol, and the salt was washed off using 75% ethanol. The RNA was then dissolved in RNase-free water. For cDNA synthesis, 1 µg of total RNA was subjected to reverse transcription conducted using the PrimeScript RT reagent kit (TAKARA Bio Inc., Kyoto, Japan) in accordance with the manufacturer’s instructions. The subsequent cDNA was analyzed using a real-time detection system (Bio-Rad Laboratories, Inc., California, USA) and the KAPA SYBR FAST One-Step qRT-PCR Kit (Kapa Biosystems, Inc., Wilmington, MA, USA) in accordance with manufacturers’ instructions. mRNA expression levels were then detected using an AZURE CIELO real-time PCR system (AZURE CIELO, Azure Biosystems, Inc., Dublin, CA, USA). Gene expression was normalized to the housekeeping genes β-actin and GAPDH and was quantified using the 2^−ΔΔCt^ method.

### IHC staining and scoring

Tissue Sects. (2 μm) were stained with primary antibodies, followed by amplification using an ABC kit (Vector Laboratories, Inc., Burlingame, CA, USA) for enhanced signal visualization. The SQSTM1/p62 primary antibody was employed. The staining intensity of SQSTM1/p62 was quantified using ImageJ software [30, 31].

### Wound healing assay

For ether-lipid treatments, the human HCC cell lines (Tong, and Huh7) were pretreated with 10 nM of ether-linked phosphatidylcholine (PC O–; 878112; Avanti Research, Alabaster, USA) and ether-linked phosphatidylethanolamine (PE O–; 878130; Avanti Research, Alabaster, USA) for 2 days. After treatment, the cells were seeded onto Culture-Insert two-well plates (80209; ibidi GmbH, Martinsried, Germany) until they reached 90% confluence. After 24 h, the Culture-Insert plates were removed, and images were captured at 0 and 24 h using a bright-field microscope.

For tranilast treatment, the HCC cells were pretreated with PC O–, combined with tranilast (200 µM; T0318, Sigma-Aldrich, Burlington, MA, USA) for 2 days. After treatment, the cells were seeded onto Culture-Insert two-well plates until they reached 90% confluence. After 24 h, the Culture-Insert plates were removed, and images were captured at 0 and 24 h using a bright-field microscope.

For the β-carotene (C4582; Sigma-Aldrich, Burlington, MA, USA) and retinoic acid (R4643; Sigma-Aldrich, Burlington, MA, USA) treatments, the human HCC cell lines were seeded onto Culture-Insert two-well plates until they reached 90% confluence. After a 24 h wait, the Culture-Insert plates were removed. Subsequently, the cells were treated with β-carotene and retinoic acid (20 µM) for another 24 h. Images were taken at both the start (0 h) and end (24 h) of this treatment by using a bright-field microscope. For combined treatments, the ether-lipid-treated cells were seeded onto Culture-Insert two-well plates. After 24 h, the Culture-Insert plates were removed, and the cells were maintained in β-carotene or retinoic acid-containing culture medium, and then images were captured at 0 and 24 h using a bright-field microscope.

For fenofibrate treatment, HCC cells were pretreated with fenofibrate (10 µM; F6020, Sigma-Aldrich, Burlington, MA, USA) for 72 h. The cells were then seeded onto Culture-Insert two well until they reached 90% confluence. After 24 h, the insert well was removed, and the cells were maintained in a culture medium, and then images were taken at 0 and 8 h. All images were analyzed using the “Wound_healing.ijm” macro in ImageJ/Fiji [32].

### Cell invasion assay

For treatment with ether-linked lipids, the HCC cells were seeded at a density of 1 × 10^5^ cells/well in the upper chamber of 24-well culture inserts filled with FBS-free DMEM medium. These inserts were coated with Matrigel (BD Bioscience, Franklin Lakes, New Jersey, USA) for 4 h. In the lower chamber, culture medium containing PC O– (10 nM) and PE O– (10 nM) was added. After a 24 h incubation, the Matrigel was removed, and the membrane was fixed with 4% formaldehyde for 30 min. The membrane was then stained with 0.05% crystal violet in 60% ethyl alcohol for 30 min, washed twice with PBS, excised, and placed onto microscope slides. Imaging was conducted using a fluorescence microscope (Nikon, ECLIPSE 80i). For the treatments and co-treatments with β-carotene (20 μM), retinoic acid (20 μM), and tranilast (200 μM), a similar protocol was followed, with the lower chamber of the 24-well culture inserts containing β-carotene, retinoic acid, or tranilast. Cell number quantification was conducted using the Image J analysis method [32, 33].

### Immunofluorescence and quantitative analysis

Tong cells were seeded onto four-well glass chamber slides (PEZGS0416, Millipore, Burington Massachusetts, USA) and treated accordingly. Cells were rinsed with PBS and fixed with 2% PFA (Macron) for 15 min at room temperature. After three PBS washes (5 min each), cells were permeabilized with 0.2% Triton X-100 (Bio Basic Inc., Markham, Ontario Canada) in PBS for 30 min. They were then washed, blocked with 1% BSA in PBST for 1 h, and incubated overnight at 4 °C with primary antibodies (1:100). After washing, cells were stained with fluorescent secondary antibody (1:200; Abcam plc, Cambridge, UK) and DAPI (1 µg/mL; Sigma-Aldrich, Burlington, MA, USA) for 1 h at room temperature. Cells were washed again, stained with 5 μg/mL Bodipy 493/503 (Invitrogen, Carlsbad, CA, USA) for 15 min, washed, and finally mounted with mounting media (Invitrogen, Carlsbad, CA, USA). Imaging was conducted using a Leica SP8 microscope (63x/1.4NA lens) or an ANDOR dragonfly high-speed confocal system (63x/1.4NA lens), capturing at least five images per well for each experiment. For immunofluorescence (IF) staining, the following antibodies were used: anti-ADFP antibody (ab108323; Abcam plc, Cambridge, UK), anti-ADRP antibody (sc-377429; Santa Cruz Biotechnology, Inc., California, USA), anti-SQSTM1/p62 antibody (ab91526; Abcam plc, Cambridge, UK), anti-LC3-B antibody (ab192890; Abcam plc, Cambridge, UK), anti-LC3 A/B antibody (Abc929; Sigma-Aldrich, Burlington, MA, USA), anti-ALDH3A2 antibody (PA5-120435; Invitrogen, Carlsbad, CA, USA), and anti-ALDH3A2 antibody (sc373921; Santa Cruz Biotechnology, Inc., California, USA).

p62 puncta number and size were measured using Fiji/ImageJ. Colocalization analysis was performed by comparing green and red channels in the same field, generating a scatterplot and applying thresholds to identify overlapping areas. The size, diameter, and number of colocalized puncta were quantified. For each experiment, over 1000 cells from 3–5 images were analyzed. Results are shown as mean ± standard deviation, and statistical significance was determined using Student’s t-test with a p-value > 95% considered meaningful.

### Luciferase reporter assay

The human HCC cells were seeded onto a 24-well plate at a density of 10^5^ cells per well. The cells were transfected with 0.5 µg of PPRE X3-TK-luc (#1015; addgene, Watertown, USA) plasmid and pRL-TK using Lipofectamine 2000 (Invitrogen, Carlsbad, CA, USA) in accordance with the manufacturer’s instructions. After 24 h of incubation, the cells were maintained in culture medium containing 20 µM PPAR ligand (either β-carotene or retinoic acid) for an additional 48 h. After 48 h, both firefly and Renilla luciferase activities were measured using the Dual-Luciferase Reporter Assay System (Promega Corporation, Madison, WI, USA) in accordance with the manufacturer’s instructions and then detected through FLUOROSKAN FL (Thermo Fisher Scientific Inc., Waltham, MA, USA) [34, 35].

### Three-dimensional invasion assay

The method was modified from published paper [36]. In brief, Preparation of cell suspension (0.5–1 × 10^4^ cells/ml) and dispense 200 µL of cell suspension to 96-well round bottom plates. Incubate the plate in incubator for four days to form tumor spheroid. Four days later, place the plate on ice and remove 100 μl of medium. Then dispense 100 μl of BMM (containing 10 ng/ml of EGF) into the well. Transfer the plate to an incubator to make the BMM solidify. One hour later, add 100 μl/well of complete growth medium containing 30 μM of Fenofibrate (3 × the desired final concentration). After treatment, take the images at different time point (0, 24 h, 48 h). All the images were quantified by using Image J.

### Isolation of G-actin and F-actin

G-actin and F-actin were isolated using a previously described method [37, 38], with some modifications. In brief, the human HCC cells were cultured in a 60-mm dish and treated with PC O– and co-treated with tranilast for 48 h. Subsequently, the cells were washed with PBS and then lysed with 0.5 mL of F-actin stabilizing buffer (comprising 50 mM PIPES, pH 6.9, 50 mM NaCl, five mM MgCl2, five mM EGTA, 5% glycerol, 0.1% Triton X-100, 0.1% Tween-20, 0.1% NP-40, 0.1% 2-mercaptoethanol, one mM ATP, and a protease inhibitor cocktail) for 15 min on ice. The cells were then scraped off and centrifuged at 16,000 × *g* for 75 min. The supernatant containing the G-actin fraction was carefully collected, and the pellet containing the F-actin fraction was resuspended in 0.5 mL of chilled water supplemented with one mM cytochalasin D (BML-T109; Enzo Biochem, Inc., NY, USA) and allowed to incubate for one h on ice. Equal volumes of the G-actin and F-actin fractions were then subjected to Western blot analysis.

### Statistics

All the results and analyses in the in vitro experiments were performed in triplicate and shown as mean ± SD. Statistical analysis was performed with Prism version 6 (GraphPad Software, San Diego, CA, United States). An unpaired Student’s t-test was used to compare two groups to determine significant differences (*p* < 0.05). Additional material and methods are described in the supplementary section (Supplementary Table S1- Table S3).

## Results

### Lipidomic and transcriptomic reprogramming in hepatocarcinogenesis

**Fig. 1:**
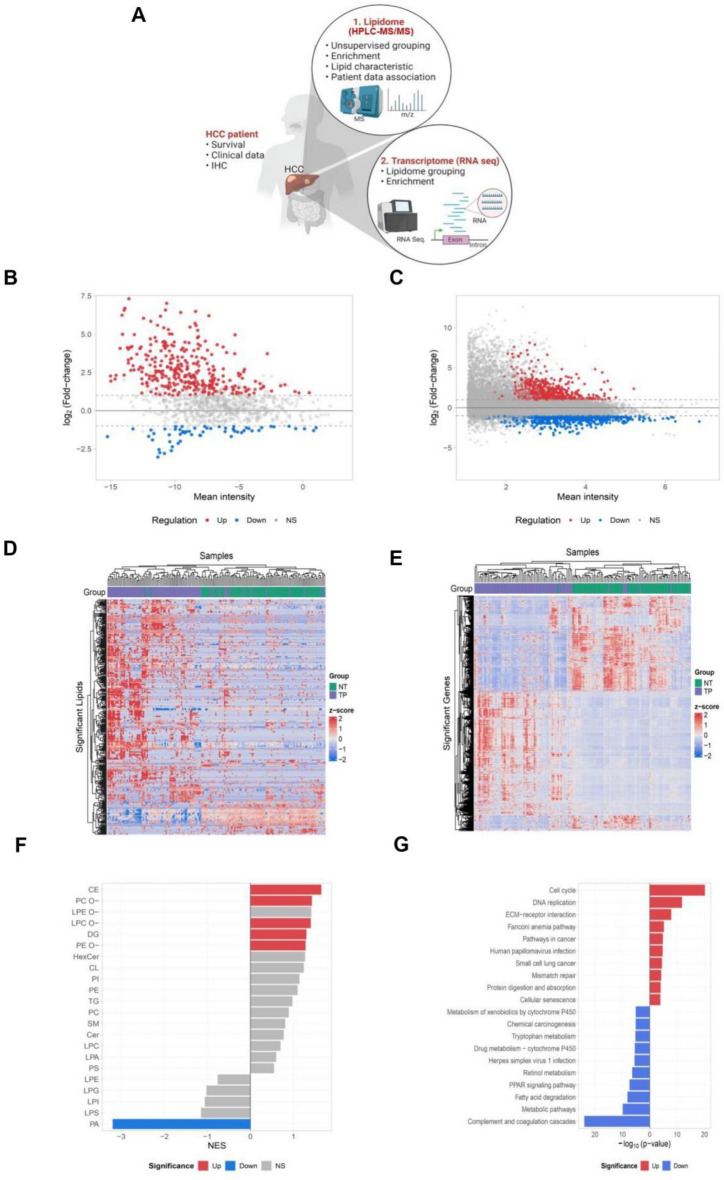
Differential lipidomic and transcriptomic profiling between normal and tumor tissues from patients with hepatocellular carcinoma (HCC). **A** Workflow diagram summarizing the collection of tissue specimens from 98 HCC patients recruited from China Medical University Hospital (CMUH) and the subsequent lipidomic and transcriptomic analyses performed. Lipidomics profiling was conducted using HPLC–MS/MS, while transcriptomic analysis was performed by RNA sequencing (RNA-seq.). **B** MA plot illustrating the differential lipid species expression between tumor and normal tissues. Significantly upregulated lipids in tumor samples are indicated in red, significantly downregulated in blue, and non-significant changes (NS) in grey. **C** MA plot depicting differential gene expression from transcriptomic analysis between tumor and normal tissues. Genes significantly upregulated in tumor samples are indicated in red, significantly downregulated genes in blue, and non-significant changes (NS) in grey. **D** Heatmap visualizing the expression patterns of significantly different lipid species across the normal tissue (NT, green) and tumor tissue (TP, purple) samples. Lipid species levels are indicated using a z-score color scale. **E** Heatmap displaying the differential expression patterns of significantly altered genes between normal and tumor samples. Gene expression levels are represented using a z-score color scale. **F** Class enrichment analysis of significantly different lipid species, highlighting lipid classes enriched in tumor versus normal tissues. Lipid classes significantly upregulated in tumor samples are shown in red, downregulated in blue, and non-significant (NS) in grey. **G** Functional enrichment analysis of significantly altered genes identified from transcriptomic profiling. Pathways significantly enriched among upregulated genes in tumor tissues are indicated in red, whereas pathways enriched among downregulated genes are depicted in blue

Tissue specimens representing normal and tumor regions were collected from a cohort of 98 consecutive hepatocellular carcinoma (HCC) patients recruited at China Medical University Hospital, Taiwan (Supplementary Figure S1). Lipidomic and transcriptomic profiling was performed to investigate associations between lipid and gene expression patterns and the clinical characteristics of HCC (Fig. 1A). Differential expression analysis, defined by an adjusted p-value < 0.05 and |log2(fold change)|> 1, identified 384 significant lipid species (Fig. 1B) and 1,453 significant genes (Fig. 1C) that distinguished tumor tissues from adjacent normal tissues (Fig. 1D–E). Lipid set enrichment analysis revealed that lipid classes such as ether-linked phosphatidylcholine [PC(O–)], ether-linked phosphatidylethanolamine [PE(O–)], cholesterol ester (CE), and ether-linked lysophosphatidylethanolamine [LPE(O–)] were significantly enriched in tumor samples (Fig. 1F), indicating their potential involvement in key metabolic pathways associated with HCC progression. Among these carcinogenesis-related lipid classes, CE accumulation is a commonly reported finding [39], whereas the enrichment of ether-lipids, including PC(O–), PE(O–), and LPE(O–), represents a relatively novel discovery in this context.

Functional enrichment analysis of significant genes identified several critical biological pathways altered in HCC. Figure 1G displays the top ten up- and down-regulated pathways based on KEGG pathway analysis. Notably, cell cycle-related pathways were among the most significantly upregulated, while complement system pathways were prominently downregulated. Importantly, lipid-related pathways, including Fatty Acid Omega Oxidation and Peroxisome Proliferator-Activated Receptor (PPAR) Signaling, were also significantly downregulated, underscoring the intricate interplay between lipid metabolism and tumor biology in HCC. The upregulated pathways are consistent with increased cell proliferation, a hallmark of cancer. However, the observed upregulation of specific lipid classes (e.g., ether-lipids) could not be fully explained by the concurrent transcriptomic changes in canonical lipid synthesis pathways.

### Lipidomic trait for HCC progression

**Fig. 2:**
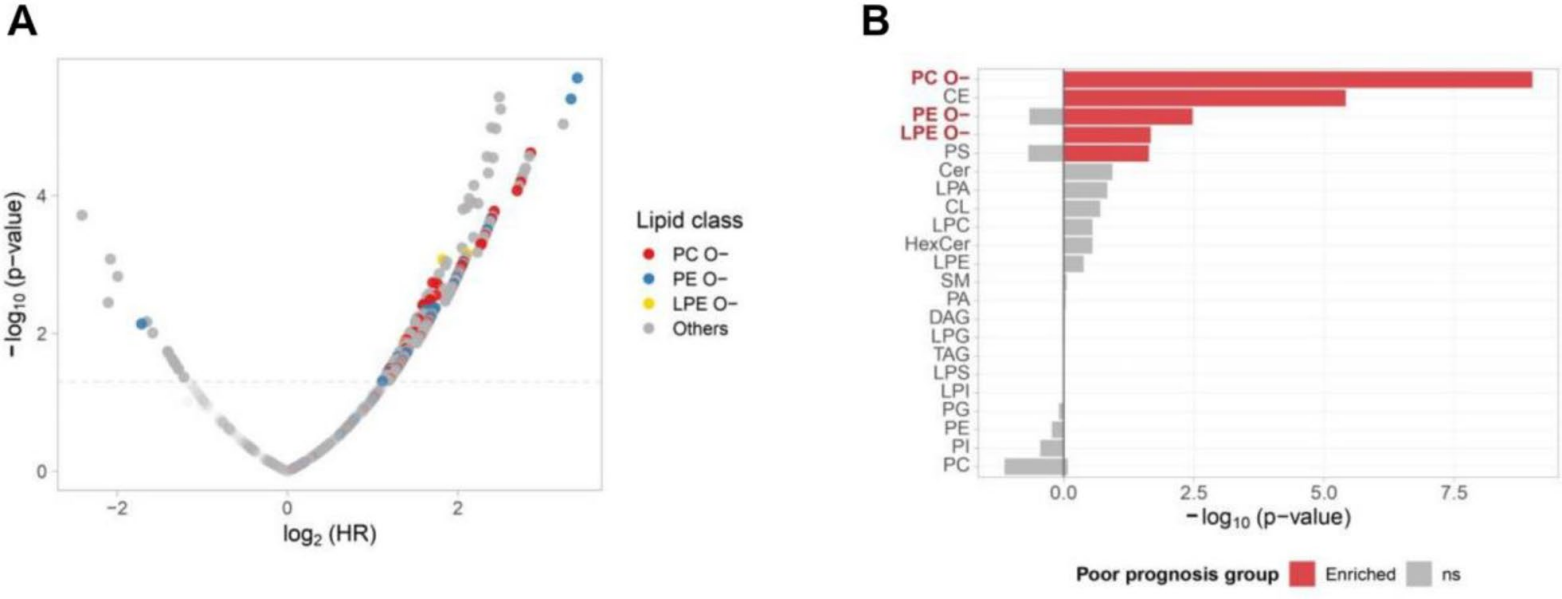
Lipid species abundance and survival analysis in HCC. **A** Volcano plot illustrating the results of survival analysis based on lipid species abundances. The x-axis represents the log_2_-transformed hazard ratios (HR), indicating the association between lipid species levels and survival outcomes, with higher values indicating poor prognosis. The y-axis shows the -log_10_ (p-value), reflecting the statistical significance of each lipid species. Specific lipid classes are highlighted in distinct colors, including PC O– (red), PE O- (blue), and LPE O– (yellow), with non-highlighted lipid classes grouped as others (gray). **B** Class enrichment analysis summarizing lipid classes significantly associated with poor prognosis based on survival analysis shown in panel A. Lipid classes enriched in the poor prognosis group are indicated in red, while non-significant (ns) lipid classes are shown in gray

Given the observed alterations in lipid metabolism, we next examined the prognostic relevance of lipid species identified in the lipidomic profile. A univariate Cox regression analysis, illustrated by a volcano plot in Fig. 2A, identified lipids significantly associated with patient survival (log-rank p-value < 0.05). Enrichment analysis of survival-associated lipid classes highlighted PC O–, CE, PE O–, and LPE O– as critical prognostic indicators (Fig. 2B), coinciding with lipid classes enriched in the tumor vs. normal differential expression analyses (Fig. 1F). Further analysis of lipid characteristics, including carbon chain length and degree of unsaturation (number of double bonds), revealed differential prognostic implications across these lipid classes (Supplementary Figure S2).

Comparison of the lipidomic changes associated with carcinogenesis (tumor vs. normal) and prognosis revealed that ether-lipids were consistently upregulated and associated with poor survival. This data suggested a master regulatory pathway, other than lipid anabolism, responsible for the abundance of ether-lipids throughout HCC development.

### Ether-lipids as dominant features of a high-risk lipidomic subtype in HCC progression

**Fig. 3:**
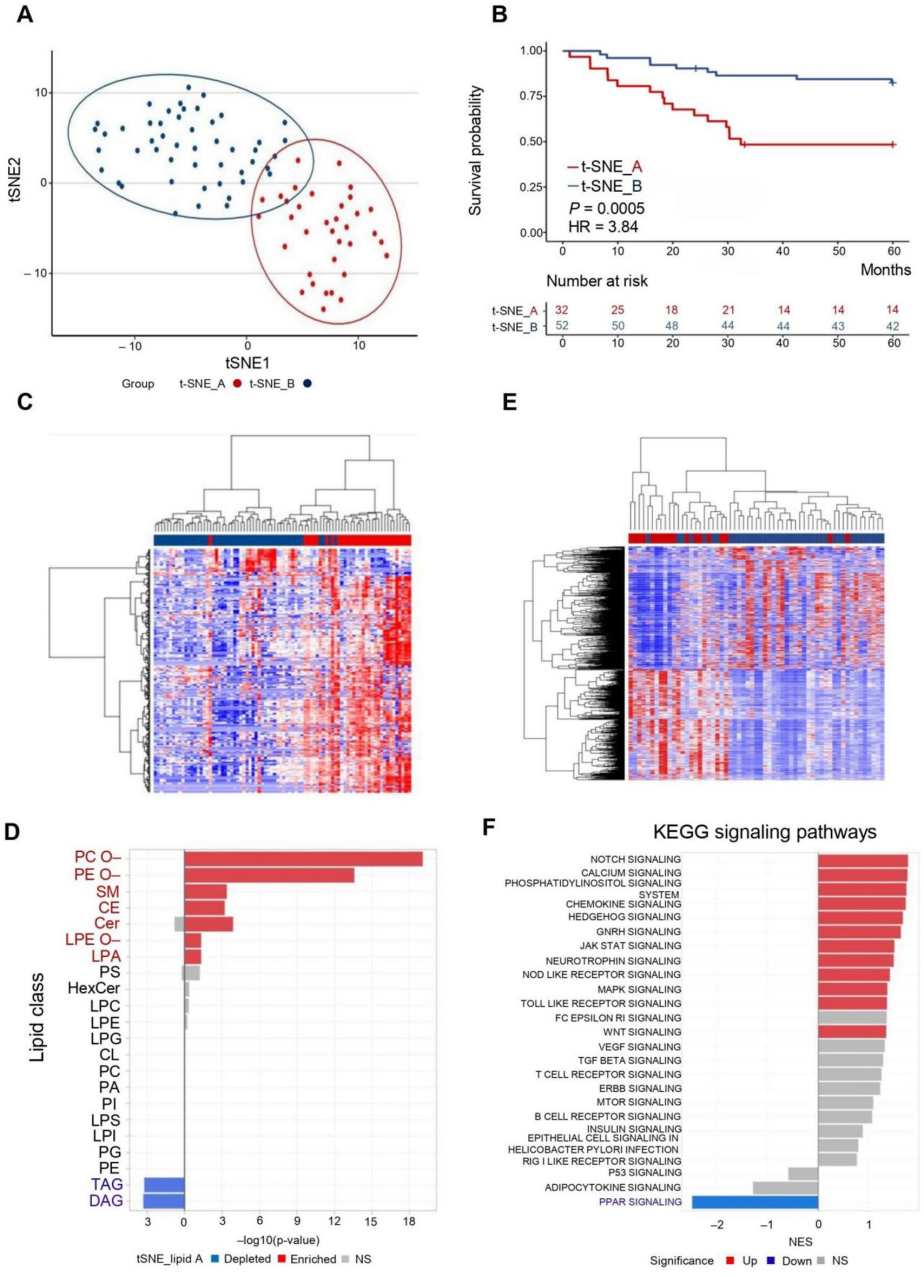
Lipidome-based classification of HCC patients. **A** t-distributed Stochastic Neighbor Embedding (t-SNE) plot depicting unsupervised clustering based on lipidomic profiles. Patients were categorized into two distinct lipidome-based subtypes: t-SNE_A (red dots) and t-SNE_B (blue dots). **B** Kaplan–Meier survival analysis comparing the overall survival between the two lipidomic subtypes identified in panel A. The survival probability over time is illustrated, demonstrating significant differences between t-SNE_A (red) and t-SNE_B (blue) groups, with log-rank test p-value indicated. **C** Heatmap illustrating the differential abundance of significant lipid species between the two lipidomic subtypes. The lipid expression is represented by a z-score color scale, with the red and blue color bars indicating the t-SNE_A and t-SNE_B groups, respectively. **D** Lipid class enrichment analysis revealing lipid classes significantly enriched or depleted in the t-SNE_A group compared to the t-SNE_B group. Classes significantly enriched in t-SNE_A are indicated in red, significantly depleted classes in blue, and non-significant (ns) classes in gray. **E** Heatmap depicting the differential gene expression patterns significantly distinguishing the t-SNE_A and t-SNE_B groups. Gene expression levels are represented by a z-score color scale, and the color bars denote the two lipidomic subtypes (red: t-SNE_A, blue: t-SNE_B). **F** KEGG signaling pathway enrichment analysis based on differentially expressed genes between the two lipidomic subtypes. Pathways significantly upregulated in the t-SNE_A group are shown in red, pathways significantly downregulated in blue, and non-significant pathways in gray

Considering the prognostic importance of lipid profiles, we explored lipidome-based subtyping of HCC patients using t-distributed stochastic neighbor embedding (t-SNE), delineating two distinct patient groups: t-SNE_A and t-SNE_B (Fig. 3A). Clinically, the t-SNE_A group exhibited more aggressive characteristics, including advanced tumor-node-metastasis (TNM) staging, higher recurrence rates, and larger tumors compared to the t-SNE_B group (Table 1). Survival analysis confirmed significantly poorer prognosis in the t-SNE_A group (Fig. 3B).

**Table 1:**
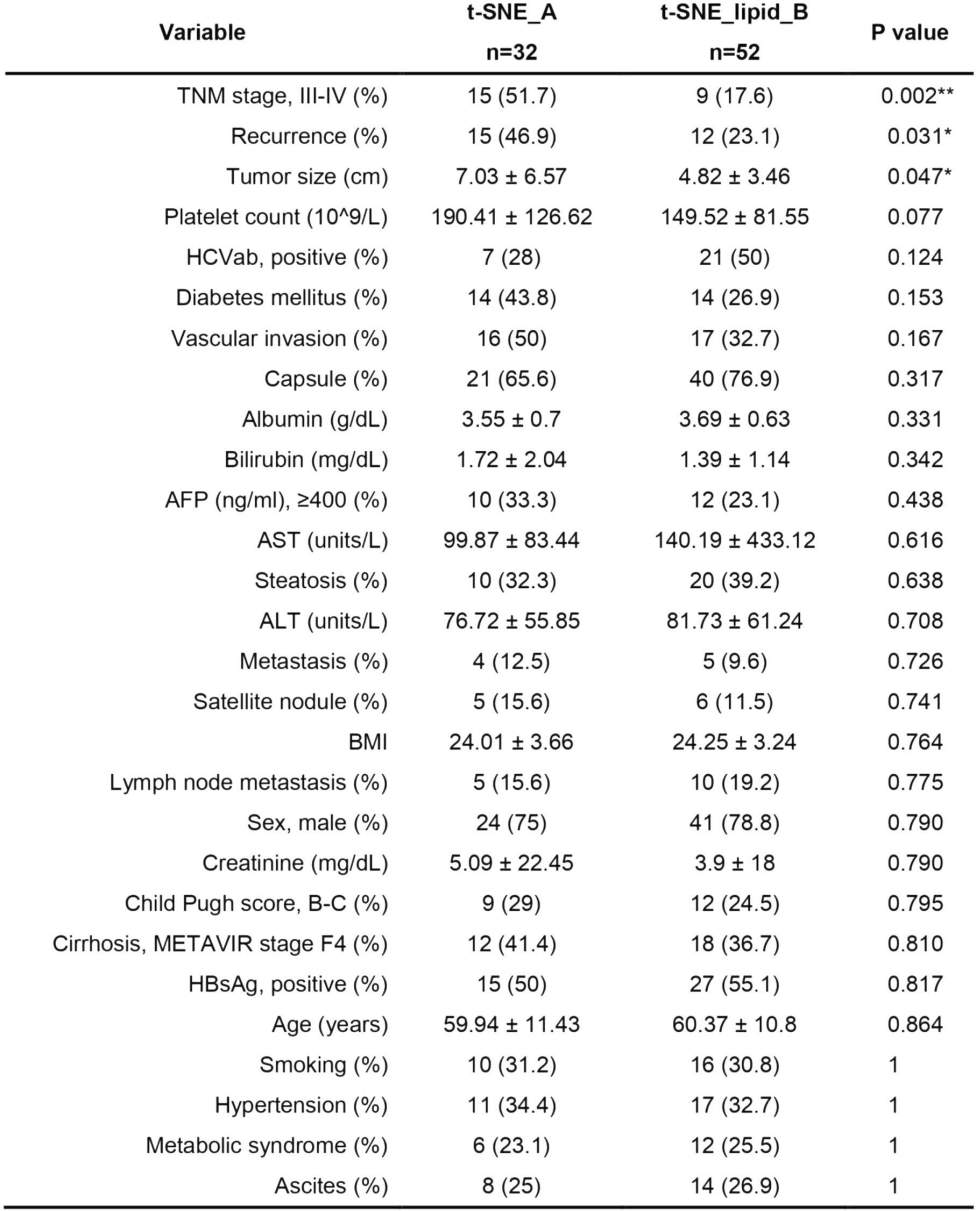
Demographic Information of Lipidome-Based HCC Subtypes

TNM: tumor, node, metastasis; HCV: Hepatitis C-Virus antibody; AST: aspartate amino transferase; BMI: Body Mass Index; AFP: alpha fetoprotein; ALT: alanine aminotransferase; METAVIR: meta analysis of histological data in viral hepatitis

Differential lipid expression analysis identified significant alterations between these groups (Fig. 3C), particularly highlighting enrichment of ether-linked phospholipids (PC O–, PE O–, LPE O–), CE, sphingolipids (sphingomyelin [SM], ceramide [Cer]), and lysophosphatidic acid (LPA) in the t-SNE_A group. Conversely, neutral lipids such as triacylglycerols (TAG) and diacylglycerols (DAG) were notably decreased (Fig. 3D). In the further analysis, a total of 159 ether-lipids and 710 non-ether-lipids were identified by lipidomic profiling; among these, 128 ether-lipids and 138 non-ether-lipids were significantly up-regulated in the t-SNE_A group. Enrichment of up-regulated species within the ether-lipid class was assessed by Fisher’s exact test (p < 2.2 × 10⁻^16^, Table 2).

**Table 2:**
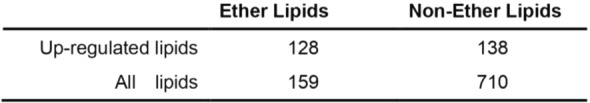
Summary of detected ether and non-ether lipid species and their up-regulation in HCC t-SNE_A versis t-SNE_B

p-value < 2.2e-16 from Fisher’s Exact test

Transcriptomic differences between the two t-SNE groups further emphasized distinct biological processes (Fig. 3E). Differential gene expression (DE) and pathway analyses using the KEGG and Reactome databases revealed significant upregulation of pathways associated with extracellular matrix (ECM) remodeling, focal adhesion, and angiogenesis in the aggressive t-SNE_A group (Supplementary Figure S3A and S3B). Conversely, pathways involved in peroxisome function, steroid hormone biosynthesis, fatty acid metabolism, and amino acid metabolism were markedly downregulated (Supplementary Figure S3C, S3D, and S3E). Notably, analysis of KEGG signaling pathways showed that the peroxisome proliferator-activated receptor alpha (PPARα) is the only significant down-regulated pathway (Fig. 3F), suggesting a critical deficit in lipid regulatory mechanisms in aggressive HCC subtypes. In addition, the PPAR signal was found consistent in carcinogenic and prognostic-related DE analysis, which suggests PPAR might be the master regulatory pathway responsible for the abundance of ether-lipids.

### Trans-Omics analyses link ether-lipids to cell mobility

**Fig. 4:**
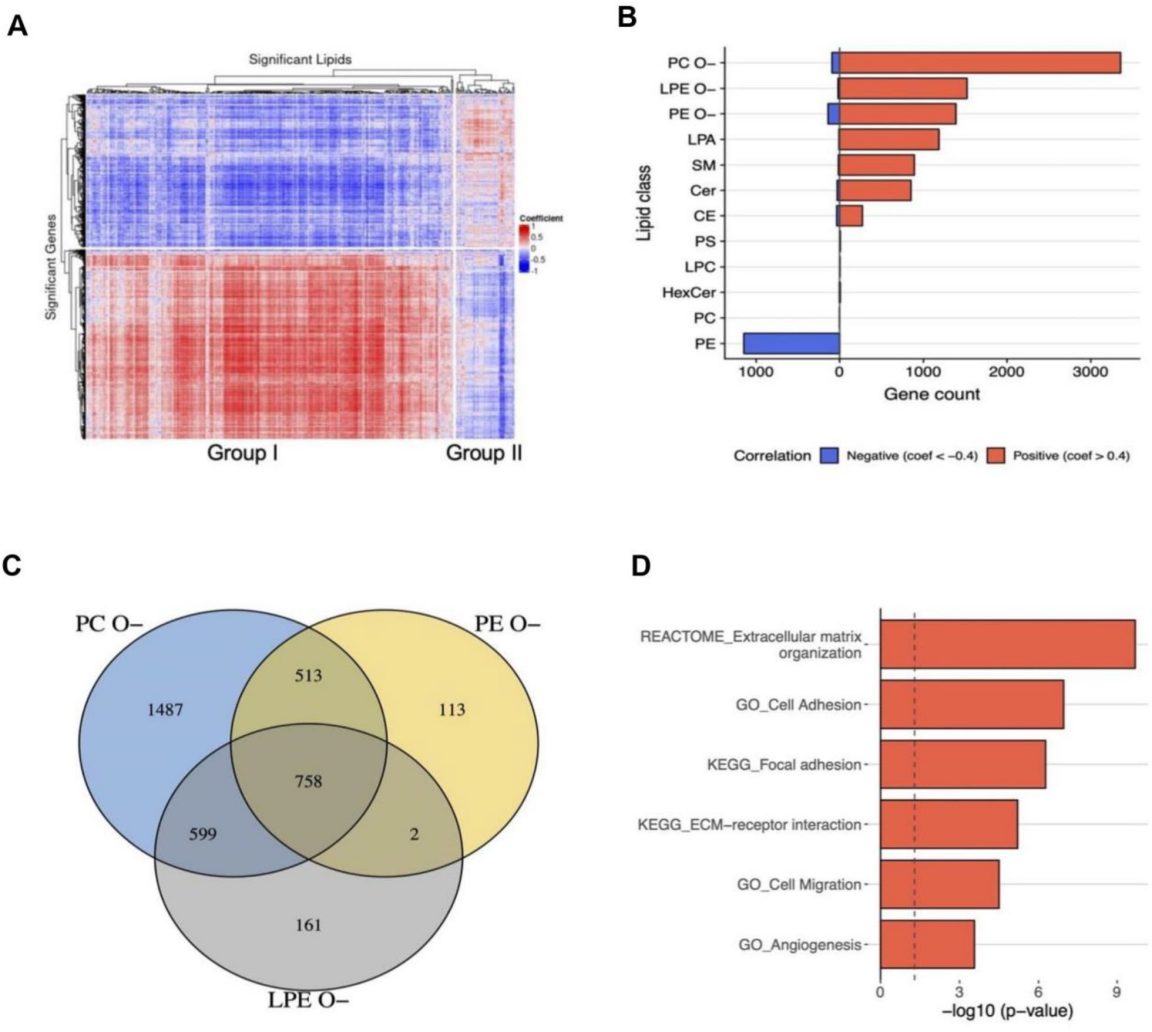
Cell mobility-related genes were associated with ether-lipids. **A** Correlation heatmap of significant genes and lipid species based on lipidomic t-SNE_A/B grouping. Group 1 and Group 2 indicate the up- and down-regulated lipid species. **B** The ranking of gene numbers that lipid classes abundance correlated with gene expressions (positive or negative correlated). The three ether-lipids, e.g., PC O–, LPE O–, and PE O–, were amongst the highest. **C** Venn diagram of three top ranked ether-lipids-genes, and found 758 genes were concordantly altered. **D** The significance ranking of the pathway enrichment analysis of 758 genes. The top three pathways were Reactome_extracellular matrix organization, GO term_Cell adhesion, and KEGG_Focal adhesion

Beyond explaining the abundance of ether-lipids, their potential roles in cancer biology are of great interest. The correlation heatmap in Fig. 4A illustrates robust associations between significant lipids and gene expression levels. Most significant lipids were positively correlated with half of the DE genes identified between the t-SNE groups. Notably, ether-linked lipids such as PC O–, LPE O–, and PE O– exhibited strong positive correlations (correlation coefficient > 0.4) with numerous genes (Fig. 4B). Subsequent pathway enrichment analysis of the 758 genes commonly associated with these ether-lipids (Fig. 4C) highlighted significant enrichment in pathways related to ECM organization, focal adhesion, cell adhesion, and angiogenesis (Fig. 4D). These results strongly support a mechanistic link between ether-lipid abundance and enhanced cell mobility, reinforcing the lipidomic influence on cancer prognosis and progression.

### PPARα downregulation increases ether-lipids to promote HCC cells migration

**Fig. 5:**
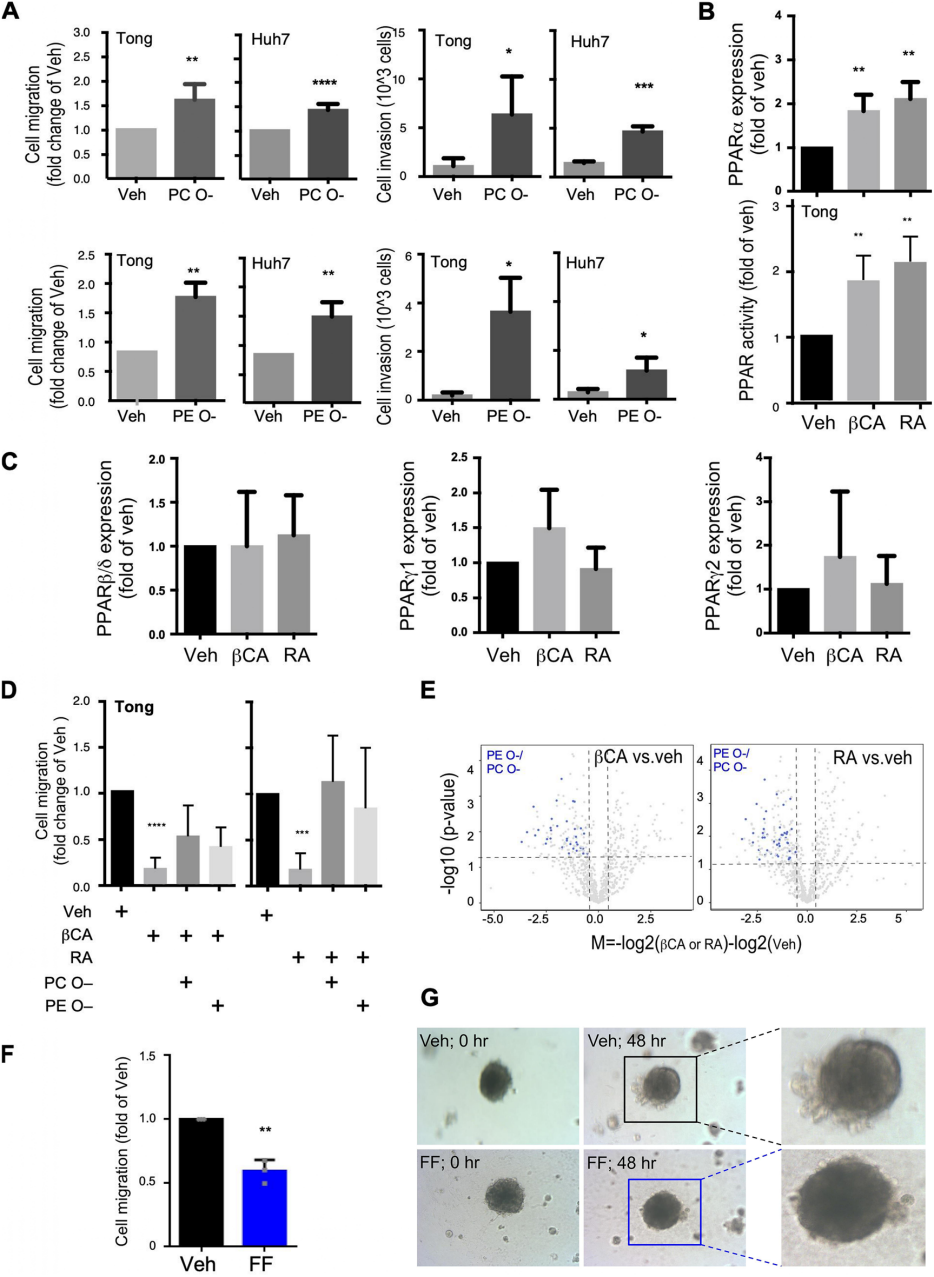
PPARα mediated ether-lipids accumulation promote cell mobility and metastasis. **A** HCC cells, specifically Tong and Huh7 cells, were treated with ether-linked phospholipids (either PC O– or PE O–; 10 nM) to evaluate their migration and invasion activities. The presented migration/invasion data were averaged from three to four independent experiments. **B** The PPARα mRNA expression (upper panel) and the PPAR promoter activities (lower panel) upon treatments of βCA and RA. **C** The PPARβ (left panel), PPARγ1 (middle panel) and PPARγ2 (right panel) mRNA expression of HCC cells upon treatments of βCA and RA. **D** Effects of PPARα agonists (βCA, left side; RA, right side; 20 μM) on suppression of HCC cell migration. Counter effects of treatments with ether-lipids (PC O– and PE O–; 10 nM) and βCA-/RA-induced cell migration are displayed. **E** βCA/RA downregulated ether-lipid abundance in HCC cells. Volcano-plot showed differential expression of lipid species, where blue-dots represents PE O– and PC O–. **F** Migration of Tong cells, as determined through a wound-healing assay 48 h after fenofibrate (FF; a PPARα agonist) treatment. **G** 3D spheroid images of filopodia with or without FF treatment. All in vitro results were derived from a minimum of three consistent experiments; * for p < 0.05, ** for p < 0.01, *** for p < 0.001, and **** for p < 0.0001

To demonstrate the cellular phenotype associated with ether-lipids, human HCC cell lines (Tong cells and Huh7 cells) were treated with PC O– and PE O–, and the data revealed that ether-lipids promoted cell migration and invasion (Fig. 5A). KEGG pathway enrichment analyses revealed that PPAR signaling was the most significantly downregulated pathway in the t-SNE_A group (Fig. 3F). PPARα expression and peroxisome activity were also consistently downregulated in this group (Supplementary Figure S3B). We next explored the relationships between PPARα and ether-lipids with cell migratory activity.

Based on the findings presented in Figs. 1, 2, 3 and 4, we hypothesized that ether-lipids promote poor prognosis and increased migration, potentially driven by PPARα downregulation. To test this hypothesis, we treated cells with β-carotene (βCA) and retinoic acid (RA), both known PPARα agonists, to model the effects of restoring PPARα activity on cellular and lipidomic phenotypes. The results showed that βCA/RA treatment predominantly upregulated PPARα expression and PPAR reporter activity (Fig. 5B), without significant impact on PPARβ/δ, PPARγ1, or PPARγ2 expression (Fig. 5C). We further observed inhibition of cell migration (Fig. 5D) and downregulation of PC O– and PE O– following βCA/RA treatment (Fig. 5E). To further test the potential of targeting PPARα to reduce metastasis, we treated HCC cells with the PPARα agonist fenofibrate. We found that fenofibrate suppressed human HCC cell migration (Fig. 5F) and reduced filopodia formation in three-dimensional (3D) spheroids derived from HCC cells (Fig. 5G). These results suggest that pharmacological activation of PPARα can mitigate migration-related phenotypes.

### PPARα downregulation impairs lipophagy, leading to ether-lipid accumulation and transient receptor potential vanilloid 2 (TRPV2)-mediated cell migration in HCC

**Fig. 6:**
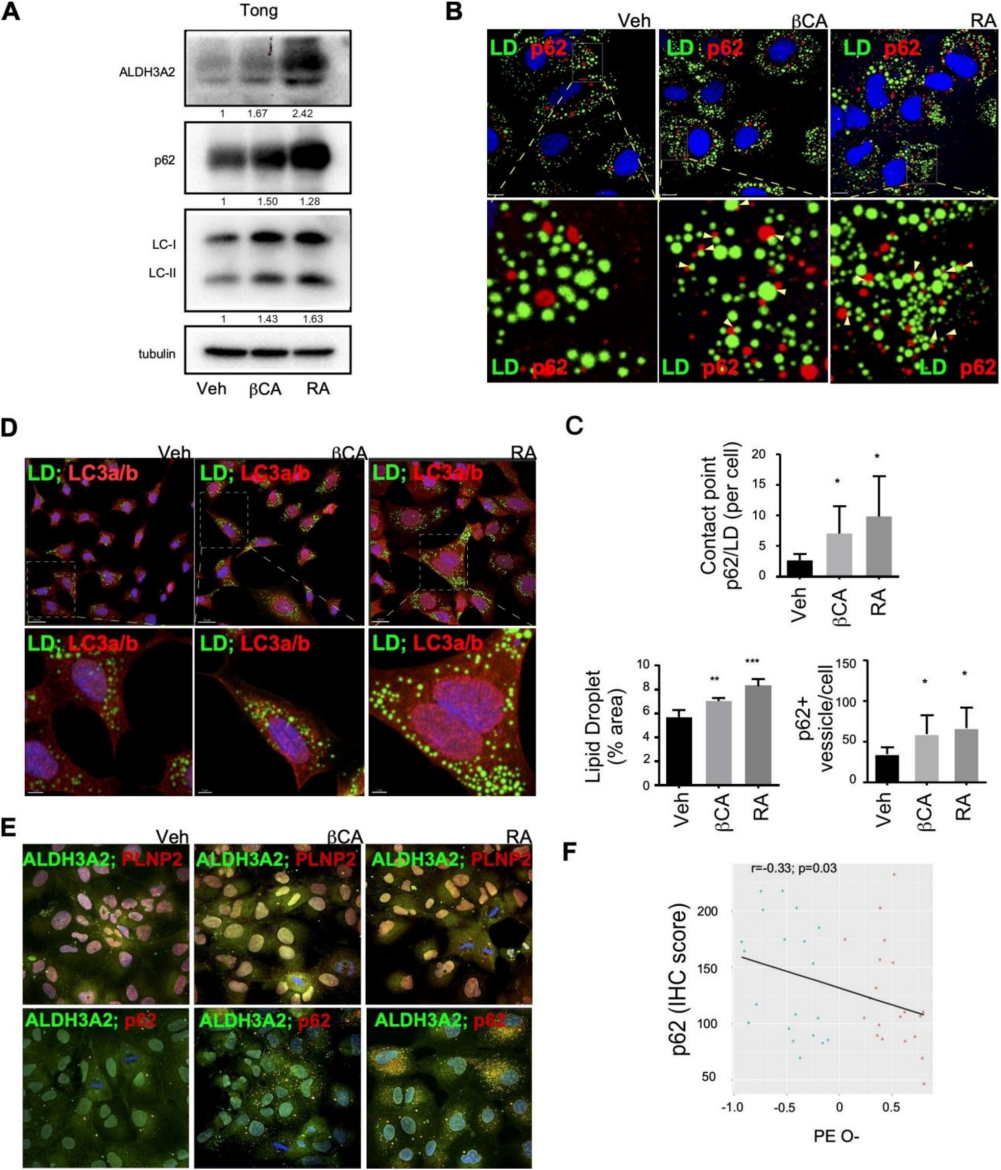
PPARα agonist promote lipophage activity in HCC cells. **A** Immunoblot analysis for ALDH3A2, p62, LC-I, and LC-II expression in HCC cells upon βCA/RA treatments. Values represent fold change relative to Veh. **B** Representative immunofluorescence images of p62 and lipid droplet in Tong cells. From left to right column images indicate as vehicle (control), β-carotene (βCA; 20 μM), and retinoid acid (RA; 20 μM). The upper panel indicated 63X magnified confocal images demonstrating the association of p62 (red) and lipid droplet (green)—scale bars: A, 10 μm. The lower panel showed enlarged images of the upper panel and zoomed in as indicated with a white dash box (see the upper panel). These images revealed that p62 puncta (red) showed less colocalization of p62 with lipid droplets (green) in the control group but a significant increase in the association of p62 with lipid droplets in PPARα agonists groups (βCA and RA). The association of p62 with lipid droplet turnover may suggest an alternative, p62-mediated lipids-clearance pathway. **C** The quantification of (B). **D** Double staining of LC3-a/b (red) and BODIPY (green) in Tong cells treated as indicated vehicle, β-carotene (βCA; 20 μM), and retinoid acid (RA; 20 μM) for 24 h. Representative confocal images of were shown. Boxed areas are enlarged (white) and showed in the lower panel. **E** Representative confocal merged images on double-staining with PLN-2 (Perilipin-2: red; upper panel) and ALDH3A2 (green) or p62(red; lower panel) and ALDH3A2 (green). The upper panel of (E) were merged confocal images with PLN-2 (red) and ALDH3A2 (green). **F** Association between IHC intensity of p62 and the expression levels of ether-lipids (PE O–). A notable inverse correlation between p62 and ether-lipid (log10-transformed abundance) was observed (R = − 0.33, p = 0.03). All in vitro results were derived from a minimum of three consistent experiments; * for p < 0.05, ** for p < 0.01, and *** for p < 0.001

Lipophagy refers to the autophagic degradation of Lipid droplets (LD), which helps maintain lipid homeostasis by consuming lipid “wastes.” The p62 (also called sequestosome-1) protein is an LD receptor that binds to autophagic marker LC3 [40] on the autophagosome for degradation [41–43]. To determine whether PPARα downregulation is a lipophagy-related event, we treated cells with βCA/RA and observed p62 and LC3 upregulation. Furthermore, aldehyde dehydrogenase three family member A2 (ALDH3A2; an enzyme for ether-lipid oxidation to fatty acids [44, 45])—a PPARα downstream target gene—was upregulated (Fig. 6A and Supplementary Figure S4). We examined the effects of βCA/RA treatment on ALDH3A2, p62, and LC-I/II expression by immunofluorescence staining and observed that these markers were differentially upregulated in the cells (Fig. 6B–E). Although βCA/RA treatment increased p62 colocalization with LD (Fig. 6B and C), no notable colocalization with LC3a/b occurred (Fig. 6D), suggesting that βCA/RA treatment may act through mechanisms distinct from those of conventional autophagy. Moreover, ALDH3A2 exhibited enhanced colocalization with perilipin-II and p62 after βCA/RA treatment (Fig. 6E), suggesting that ALDH3A2-containing lipophagosomes target LD reservoirs (26) for ether-lipid consumption. These findings indicate that PPARα modulates ether-lipid levels by enhancing their clearance, thereby influencing cell migration.

PPARα expression might be associated with lipid removal from LDs through lipophagy, followed by lipid exportation. Accordingly, we analyzed specimens collected from our patient cohort to determine the association between PC O–/PE O– abundance and immunohistochemical staining scores for p62 expression. Higher PC O–/PE O– levels were associated with lower p62 expression (Fig. 6F).

**Fig. 7:**
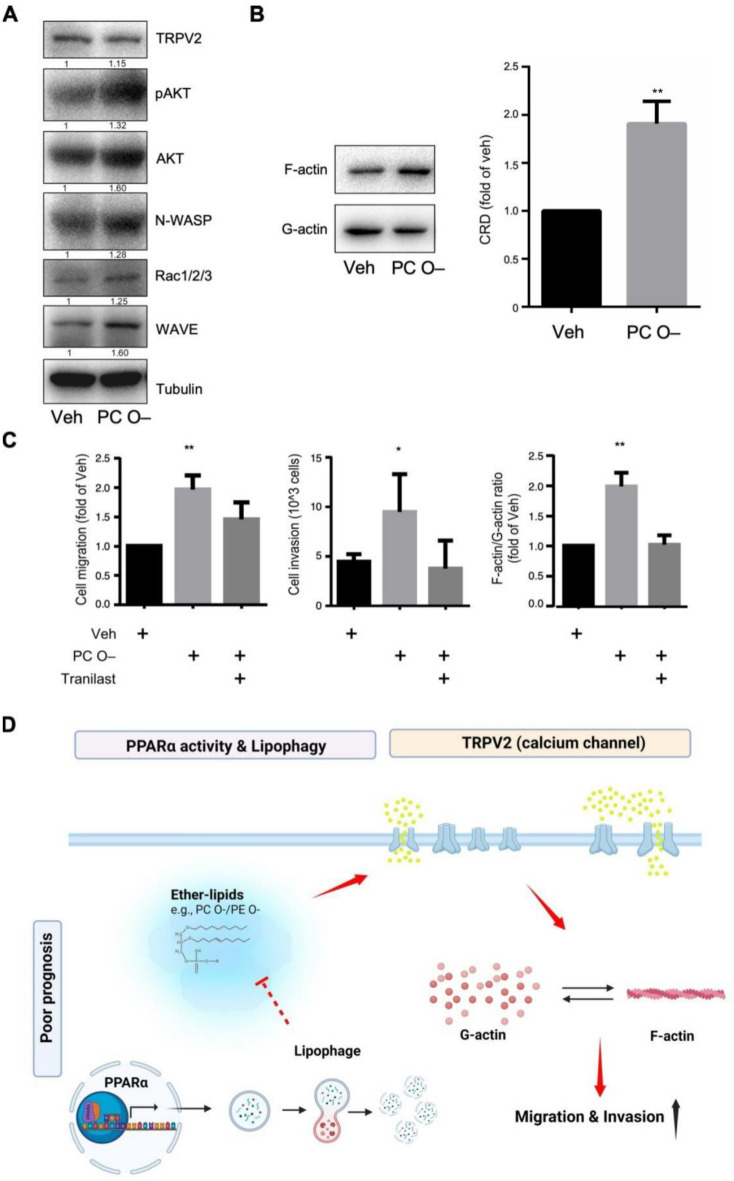
Ether-lipids promote HCC prognosis through TRPV2-mediated cytoskeletal reorganization. **A** The calcium channel protein TRPV2, and the cytoskeletal rearrangement signals, e.g., AKT, N-WASP, Pac1/2/3, and WAVE were detected by immunoblot. Values represent fold change relative to Veh. **B** Cytoskeletal rearrangement degree (CRD; F-actin/G-actin ratio) was increased upon PC O– treatment. Left-hand side panel: representative immunoblots of F-actin or G-actin abundance in Tong cells upon vehicle or PC O– treatments. Right-hand side panel: Quantitation result of CRD from three individual set of experiments. The Y-axis represent the values normalized with vehicle treatments. **C** Cellular phenotypes, such as cell migration (left-hand side), invasion (middle), and cytoskeletal reorganization capacity (right side), induced by PC O– could be reversed by tranilast (200 uM) cotreatment. **D** Schematic representation of the study indicating that PPARα mediate diminished lipid scavenging by lipophagy, trigger the ether-lipids accumulation and further mediate TRPV2 channel to activate cell migration, and finally leading to poor prognosis

A study reported that ether-lipids enhance cell migration, possibly through TRPV2 (a calcium channel protein) and its downstream signaling [46]. Although TRPV2 expression was largely unaffected by PC O–, we observed a general upregulation in cytoskeletal-related signaling pathways such as AKT, N-WASP, Rac1/2/3, and WAVE (Fig. 7A and Supplementary Figure S5). Indeed, the F-actin/G-actin ratio was increased in PC O– treatment (Fig. 7B). This observation suggests that PC O– promotes cytoskeletal reorganization primarily through TRPV2 activity. To verify this hypothesis, we applied tranilast—a TRPV2 inhibitor—and observed that it nullified the promigratory (Fig. 7C, left panel), proinvasive (Fig. 7C, middle panel), and cytoskeletal reorganization (Fig. 7C, right panel) effects of dietary PC O–. In summary, ether-lipids promote cytoskeletal reorganization and cell mobility through TRPV2 activity.

Overall, our results suggest that PPARα downregulation in HCC cells can reduce lipid scavenging through lipophagy, thereby increasing ether-lipid abundance and promoting cell migration via TRPV2 channel (Fig. 7D).

## Discussion

Advancements in mass-spectrometry-based lipid profiling technology (e.g., LC–MS/MS) have facilitated the discovery of crucial lipid metabolites involved in physiological and disease states. Studies have identified specific lipid metabolites such as CE, Cer, PS, LPA, and SM as altered in different types of cancer, including breast cancer, ovarian cancer, and pancreatic cancer [47]. However, the prognostic significance of the lipidome in HCC remains unclear. This study integrates lipidomic and transcriptomic data to systematically evaluate the prognostic value of lipid metabolites in HCC, emphasizing the potential of multi-omics approaches to uncover cancer prognostic markers and improve patient management. We discovered that specific cancer lipid metabolites serve as prognostic markers by using high-throughput multi-omics analyses, and delineated the differential effects of mRNA and lipid expression on cancer prognosis.

### Ether-lipids scavenging deficit via compromised PPARα as pivotal prognostic marker

Among the prognostic significant lipid classes, ether-lipids were the most dominant. We demonstrated the role of ether-lipids in enhancing cell mobility, notably through the activation of TRPV2 and the rearrangement of actin filaments. Furthermore, our data suggest that the abundance of ether-lipids may be attributed to downregulated PPARα signaling.

Lipid droplets (LDs) dynamics and lipophagy are closely related to cellular lipid homeostasis including lipid storage, lipid degradation, and energy mobilization. LDs are dynamic organelles responsible for neutral lipid storage, while lipophagy, a selective form of autophagy, mediates LD degradation to release free fatty acids for mitochondrial β-oxidation [48, 49].

Recently, a lot of evidence has demonstrated a close association between peroxisomes and lipid droplets in various mammalian cells. For example, in Cos-7 and HepG2 cells, live-cell imaging revealed that peroxisomes formed tubular and reticular clusters in close proximity to lipid droplets [49]. Similarly, in 3T3-L1 adipocytes and mouse epididymal white adipose tissue, immunogold labeling for the peroxisomal enzyme catalase showed numerous small, dumbbell-shaped peroxisomes surrounding lipid droplets [50]. Furthermore, recent bimolecular fluorescence complementation assays have identified direct protein–protein interactions between multiple lipid droplet-associated proteins and peroxisomal marker proteins [51].

LDs are mainly cytoplastic organelle, where they can interact with endoplasmic reticulum (ER) and peroxisomes, allowed them to store various bipolar lipids, including ether-lipids [52–54]. Notably, the ether-lipids are synthesized in the peroxisomes and further modification in ER [55]. Ether lipids play essential roles in maintaining membrane architecture, mediating antioxidant defense, and modulating signaling pathways. Their biosynthesis and metabolism involve a tightly coordinated interplay among multiple organelles. The initial steps of ether lipid synthesis occur in peroxisomes, where glyceronephosphate O-acyltransferase (GNPAT) and AGPS catalyze the formation of the ether bond, while fatty acyl-CoA reductases (FAR1/2) produce fatty alcohols at the cytosolic face of the peroxisomal membrane [56]. These intermediates are subsequently transferred to the ER, where acyl chain remodeling and desaturation take place, yielding mature ether-linked glycerophospholipids such as plasmalogens. Moreover, membrane contact sites (MCSs) between LDs and peroxisomes have been identified as key hubs for the trafficking and regulation of ether lipids, allowing peroxisome-derived lipids to be stored or selectively mobilized within LDs [57]. LDs may undergo degradation via lipophagy through interactions with lysosomes, potentially contributing to the turnover and recycling of ether lipids, although direct experimental evidence remains limited.

PPARα have been reported to regulate genes related to lipophagy that specifically targets lipid droplets (LDs) for degradation [58, 59]. Regarding the curiosity of increased abundance of ether-lipids, we revealed PPARα downregulation as the bona fide mechanism. Notably, PPARα downregulation also reduced the colocalization between LDs, peroxisomes, and ER (Fig. 6). This disruption in organelle interactions may hinder ether-lipid turnover via lipophagy, leading to their accumulation which explain how ether-lipid homeostasis is linked to lipophagy and PPAR activity.

In addition to the observed downregulation of lipid anabolism in both the KEGG and Reactome analyses, consistent reductions in PPARα and peroxisome activity were observed (Fig. 3D and F). Nevertheless, lipid metabolism due to PPARα downregulation merits attention. Furthermore, our analyses of the enrichment of specific lipid-related subpathways demonstrated significant downregulation of peroxisomal lipid metabolism, DAG/TAG acyl chain remodeling, cholesterol biosynthesis mediated by sterol regulatory element-binding protein, lipoprotein remodeling, and TAG biosynthesis (Supplement Figure S3E). In summary, our transcriptomic comparison of the t-SNE_A and t-SNE_B groups indicated that a prognostic lipidome is associated with increased cellular mobility but decreased lipid anabolic or catabolic activity. Therefore, we need to investigate further the possibility of ether-lipids operating through lipid importation from the diet. This raises the possibility that ether-lipid accumulation might also be influenced by factors such as altered lipid uptake or importation.

PPARα belongs to the family of ligand-activated transcription factors and functions as a regulator of lipid, glucose, and amino acid metabolism for energy homeostasis. The regulation of PPARα in cancer progression is controversial, as it can act either as a tumor inducer or suppressor in specific cancer cell types [60]. A study using a DEN-induced hepatocarcinogenesis model in PPARα-/- mice revealed that PPARα inhibits cell proliferation and increases apoptosis according to the immunohistochemistry analysis. PPARα was also shown to suppress cancer cell growth through NF-kB and IkBα signaling pathway in vitro [61]. The other study reported that low PPARα expression is associated with clinical features such as tumor size, TNM stage, and distant metastasis in HCC. Patients with low PPARα expression also had poor survival in HCC [62]. Most studies indicate that PPARα regulates lipid catabolism-related genes and mediates its downstream signal pathway, thereby promoting cancer progression [8]. However, the mechanisms by which PPARα directly mediates intracellular lipid metabolism remain unclear. Autophagy is a cellular process that degrades dysfunctional proteins and unwanted organels to maintain cell health and homeostasis [63]. Selective autophagy is a type of autophagy which can target specific cargo by receptor including lipophagy and p62 is a classical selective autophagy receptor [64]. While p62 accumulation often indicates impaired autophagic flux [65], recent studies suggest that p62 also serves as a LDs cargo receptor in lipophagy, and its levels may increase upon enhanced lipid cargo targeting [43]. Furthermore, PPARα agonist treatment increased p62 colocalization with LDs (Fig. 6B, C), supporting active recruitment of p62 in the context of lipophagy rather than defective autophagy. This aligns with studies suggesting differential roles of p62 in lipophagy compared to general autophagy. Hence, this study found that ether-lipids promote cancer cell migration potentially via PPARα downregulation, leading to impaired lipophagy, thus blocking lipid clearance and resulting in ether-lipid accumulation.

### Ether-lipids Activates TRPV2 to promote cancer metastasis

Ether-lipids belong to the class of glycerophospholipids and exist in two forms: alkyl-acylphospholipids and alkenyl-acylphospholipids, also known as plasmalogens. Ether-lipids are synthesized in the peroxisome and completed in the ER. They are major components of cell membranes, influencing membrane dynamics and processes such as cholesterol transport. In addition to their structural role in membranes, ether-lipids are also involved in cell signaling and differentiation. Alterations in ether-lipid levels are implicated in many diseases, such as neurological disorders, metabolic disorders, and cancers [55]. In cancer biology, ether-lipids can regulate several ion channels and promote cell migration and metastasis. For example, ether-lipids could increase SK3 expression (a potassium channel), leading to calcium entry and further promoting migration and invasion. Although some studies have elucidated the function of ether-lipids in regulating ion channels in cancers such as breast, lung, prostate, and gastric cancer [66]. TRPV2 is a member of transient receptor potential (TRP) channels, belonging to the vanilloid subfamily. It is a polymodal signal integrator that activates cellular signal pathways via non-selective cation channel. TRPV2 can be activated by heat (> 52 °C), ligands such as cannabidiol, mechanical stresses, as well as lipids such as lysophospholipids and ether-lipids [67]. TRPV2 is located in the endoplasmic reticulum compartment under unstimulated conditions. Upon stimulation, activation of phosphatidylinositol 3-kinase (PI3K) is induced, leading to the translocation of TRPV2 to the plasma membrane, where it can mediate calcium influx [68]. The calcium influx in turn activate downstream signal, including AKT and cytoskeletal rearrangement promoting cancer migration, invasion, and proliferation. TRPV2 overexpression has been linked to cell proliferation, migration, and survival in some cancer including breast cancer prostate cancer, esophageal squamous cell carcinoma [46, 69, 70]. In liver cancer, high expression of TRPV2 is association with poor prognosis in HCC patient [71]. The other study indicated that TRPV2-mediated calcium influx promotes HCC progression by activating AKT signaling and enhancing apoptosis resistance [72]. However, the role and underlying mechanisms of TRPV2 in HCC was not explored previously. Here, we demonstrated that ether-lipids may enhance cell mobility through calcium signaling (Figs. 4D and 7C). TRPV2 expression was consistent across various analyses at protein levels, with or without ether-lipid treatment (Fig. 7A and Supplementary Figure S5). However, tranilast could block dietary-ether-lipid induced mobility (Fig. 7B), which is consistent with research [46] describing TRPV2 activation by plasmalogens (PE O − ; 16:0; 18:1) in breast cancer cells. This result suggests that TRPV2 activation may be related to membrane fluidity rather than gene expression changes [73].

## Conclusion

This study demonstrated that ether-lipids accelerate cancer cell mobility through TRPV2 activation and that pathological PPARα downregulation compromised ether-lipid clearance in HCC, posing significant challenges to cancer prognosis.

## Supplementary Information

Supplementary Material 1.

## Data Availability

Transcriptome data in this study are publicly available in Gene Expression Omnibus (GEO) at GSE242315.

## Abbreviations

C: Cholesterol
CE: Cholesterol ester
Cer: Ceramide
CL: Cardiolipin
CMUH: China Medical University Hospital
Cox-PH: Cox proportional hazards
DAG: Diacylglycerol
ER: Endoplasmic reticulum
FC: Free cholesterol
FF: Fenofibrate
GO: Gene ontology
HCC: Hepatocellular carcinoma
HexCer: Hexosylceramide
HR: Hazard ratios
IHC: Immunohistochemical
LD: Lipid droplet
LPA: Lyso-phosphatidate
LPC: Lyso-phosphatidylcholine
LPE O–: Ether lysophosphatidylethanolamine
LPE: Lyso-phosphatidyl-ethanolamine
LPG: Lyso-phosphatidylglycerol
LPI: Lyso-phosphatidylinositol
LPL: Lipoprotein lipase
LPS: Lyso-phosphatidylserine
NES: The normalized enrichment score
Padj: Adjusted p-value
PA: Phosphatidate
PC O–: Ether phosphatidylcholine
PC: Phosphatidylcholine
PE O–: Ether phosphatidylethanolamine
PE: Phosphatidylethanolamine
PG: Phosphatidylglycerol
PI: Phosphatidylinositol
PL: Phospholipids
PPARα: Peroxisome proliferator-activated receptor α
PS: Phosphatidylserine
RA: Retinoic acid
SM: Sphingomyelin
TAG: Triacylglycerol
TG: Triglyceride
TRPV2: Transient receptor potential vanilloid 2
βCA: β-carotene
