# Correction: Ether-lipids accumulation promotes hepatocellular carcinoma progression linked to PPARα deficiency

**DOI:** 10.1186/s12929-026-01223-4

**Published:** 2026-03-15

**Authors:** Pei-Yin Liao, Wen-Jen Lin, Pei-Chun Shen, Cian-Ru Yang, Ying-Chun Yu, Chun-Chieh Yeh, Long-Bin Jeng, Hsieh-Chou Lai, Wei-Chung Cheng, Wen-Lung Ma

**Affiliations:** 1 Graduate Institute of Biomedical Sciences, School of Medicine, China Medical University, Taichung, 406040 Taiwan; 2 Department of Medical Research, Organ Transplantation Center, China Medical University Hospital, Taichung, 404327 Taiwan; 3 Ph.D. Program for Health Science and Industry, Center of Tumor Biology, School of Medicine, China Medical University, Taichung, 406040 Taiwan; 4 Department of Gastroenterology and Department of Surgery, China Medical University Hospital, Taichung, 404327 Taiwan; 5 Cancer Biology and Precision Therapeutics Center, Ph.D. Program for Cancer Molecular Biology and Drug Discovery, China Medical University, Taichung, 406040 Taiwan; 6 Office of Research and Development, Asia University, Taichung, 413505 Taiwan; 7 Education Center, National Cheng Kung University Hospital, Tainan, 704302 Taiwan; 8 College of Medicine, National Cheng Kung University, Tainan, 701 Taiwan

**Correction: Journal of Biomedical Science (2025) 32:89** 10.1186/s12929-025-01178-y

After publication of this article [1], it was brought to our attention that figure 5 is incorrect.

The incorrect Fig. 5:

**Fig. 5:**
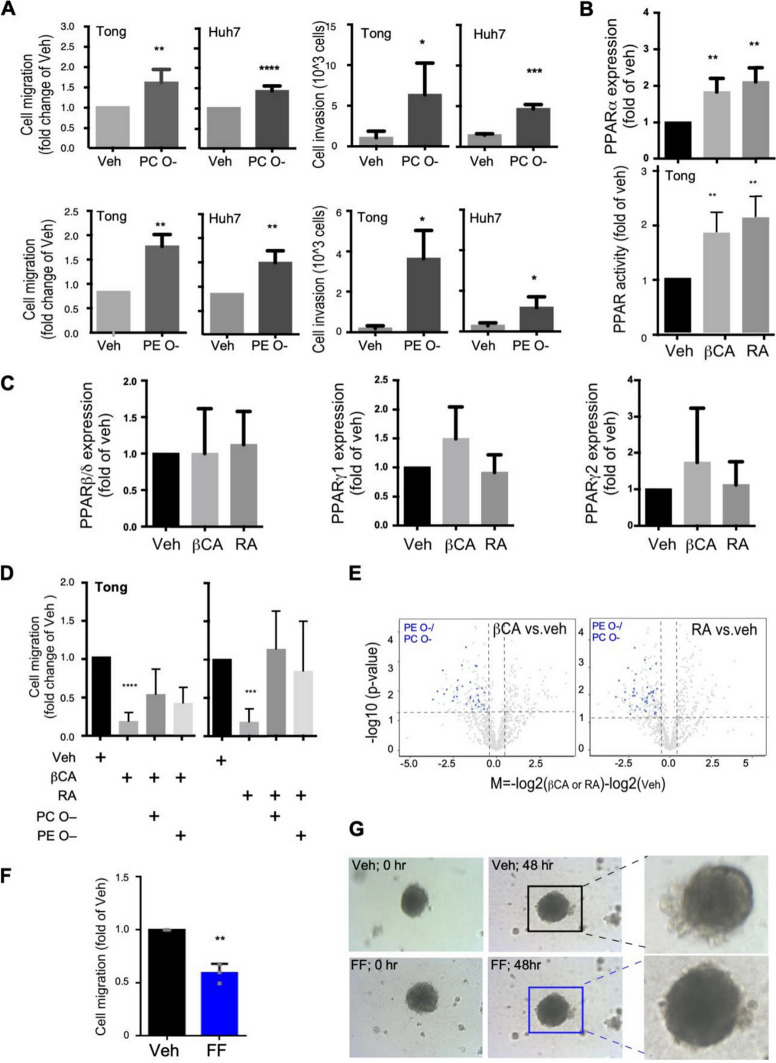
PPARα mediated ether-lipids accumulation promote cell mobility and metastasis. **A** HCC cells, specifically Tong and Huh7 cells, were treated with ether-linked phospholipids (either PC O– or PE O–; 10 nM) to evaluate their migration and invasion activities. The presented migration/invasion data were averaged from three to four independent experiments. **B** The PPARα mRNA expression (upper panel) and the PPAR promoter activities (lower panel) upon treatments of βCA and RA. **C** The PPARβ (left panel), PPARγ1 (middle panel) and PPARγ2 (right panel) mRNA expression of HCC cells upon treatments of βCA and RA. **D** Effects of PPARα agonists (βCA, left side; RA, right side; 20 μM) on suppression of HCC cell migration. Counter effects of treatments with ether-lipids (PC O– and PE O–; 10 nM) and βCA-/RA-induced cell migration are displayed. **E** βCA/RA downregulated ether-lipid abundance in HCC cells. Volcano-plot showed differential expression of lipid species, where blue-dots represents PE O– and PC O–. **F** Migration of Tong cells, as determined through a wound-healing assay 48 h after fenofibrate (FF; a PPARα agonist) treatment. **G** 3D spheroid images of filopodia with or without FF treatment. All in vitro results were derived from a minimum of three consistent experiments; * for p < 0.05, ** for p < 0.01, *** for p < 0.001, and **** for p < 0.0001

The correct Fig. 5:

**Fig. 5:**
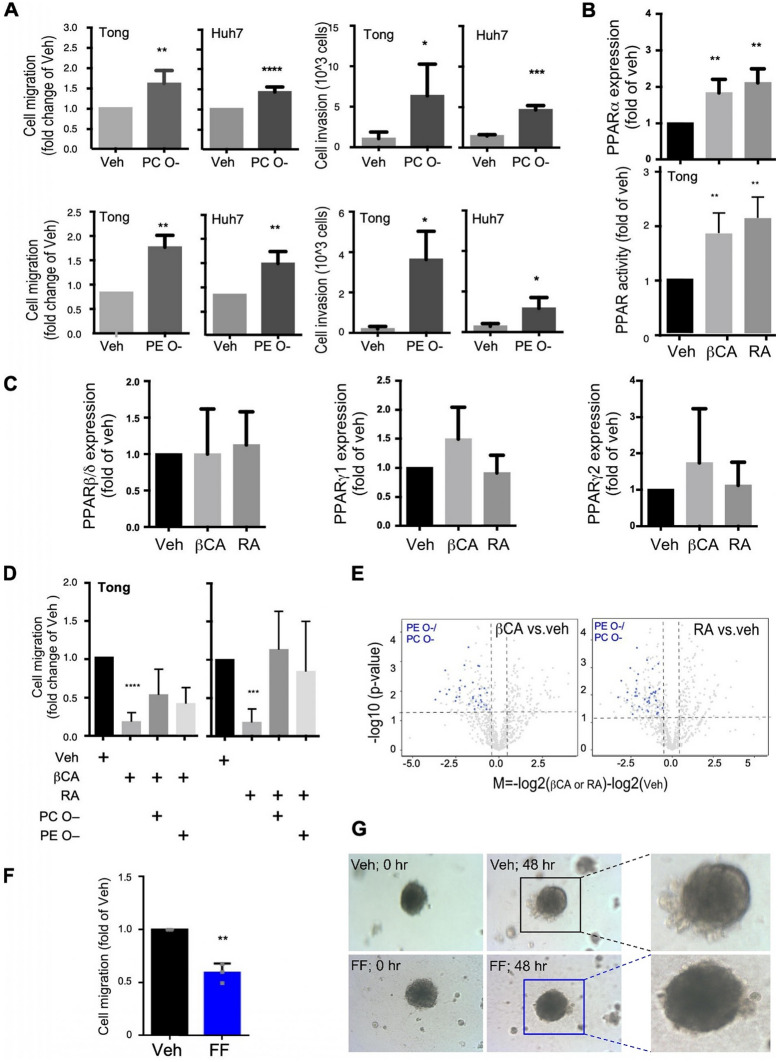
PPARα mediated ether-lipids accumulation promote cell mobility and metastasis. **A** HCC cells, specifically Tong and Huh7 cells, were treated with ether-linked phospholipids (either PC O– or PE O–; 10 nM) to evaluate their migration and invasion activities. The presented migration/invasion data were averaged from three to four independent experiments. **B** The PPARα mRNA expression (upper panel) and the PPAR promoter activities (lower panel) upon treatments of βCA and RA. **C** The PPARβ (left panel), PPARγ1 (middle panel) and PPARγ2 (right panel) mRNA expression of HCC cells upon treatments of βCA and RA. **D** Effects of PPARα agonists (βCA, left side; RA, right side; 20 μM) on suppression of HCC cell migration. Counter effects of treatments with ether-lipids (PC O– and PE O–; 10 nM) and βCA-/RA-induced cell migration are displayed. **E** βCA/RA downregulated ether-lipid abundance in HCC cells. Volcano-plot showed differential expression of lipid species, where blue-dots represents PE O– and PC O–. **F** Migration of Tong cells, as determined through a wound-healing assay 48 h after fenofibrate (FF; a PPARα agonist) treatment. **G** 3D spheroid images of filopodia with or without FF treatment. All in vitro results were derived from a minimum of three consistent experiments; * for p < 0.05, ** for p < 0.01, *** for p < 0.001, and **** for p < 0.0001

The original article has been updated.
