# Supplementary material for: Ether-lipids accumulation promotes hepatocellular carcinoma progression linked to PPARα deficiency
Source: J Biomed Sci. 2025 Sep 11;32:89. doi: 10.1186/s12929-025-01178-y (PMC12427100; doi:10.1186/s12929-025-01178-y)
Supplement: Supplementary file 1 — Supplementary Material 1. [file 12929_2025_1178_MOESM1_ESM.docx]

**Ether-Lipids Accumulation Promotes Hepatocellular Carcinoma Progression Linked to PPARα Deficiency**

**Authors:** Pei-Yin Liao^1,2^†, Wen-Jen Lin^1^†, Pei-Chun Shen^3^, Cian-Ru Yang^3^, Ying-Chun Yu^2^, Chun-Chieh Yeh^2,4^, Long-Bin Jeng^2,4^, Hsieh-Chou Lai^4^, Wei-Chung Cheng^1,3,5^‡, Wen-Lung Ma^1,2,3*^‡

**Affiliations:**

^1^Graduate Institute of Biomedical Sciences, School of Medicine, China Medical University, Taichung, Taiwan, 406040.

^2^Department of Medical Research, Organ Transplantation Center, China Medical University Hospital, Taichung, Taiwan, 404327.

^3^Ph.D. Program for Health Science and Industry, Center of Tumor Biology, School of Medicine, China Medical University, Taichung, Taiwan, 406040.

^4^Department of Gastroenterology and Department of Surgery, China Medical University Hospital, Taichung, Taiwan, 404327.

^5^Cancer Biology and Precision Therapeutics Center, Ph.D. Program for Cancer Molecular Biology and Drug Discovery, China Medical University, Taichung, Taiwan, 406040.

**Additional information:**

*Corresponding author: Wen-Lung Ma, Ph.D., maverick@mail.cmu.edu.tw; and Wei-Chung Cheng, Ph.D., wccheng@mail.cmu.edu.tw.

† These authors contributed equally to this work as co-first authors

‡ These authors contributed equally to this work as co-corresponding authors


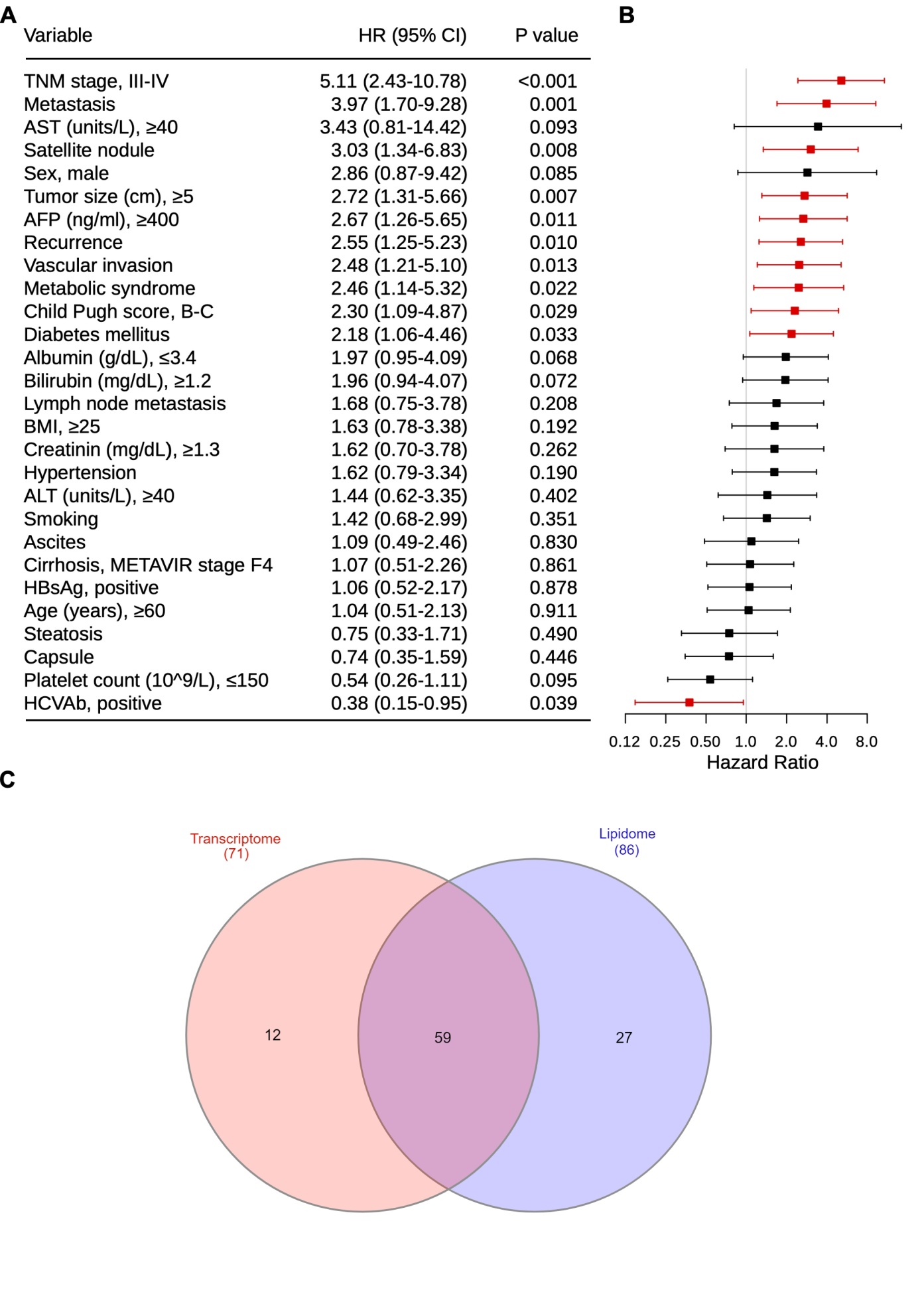


**Supplementary Figure S1. The CMUH-HCC cohort patient demography.** **(A, B**). Clinical features association to risks of death (overall survival time; hazard ratio, HR), including TNM staging (III~IV)…etc. Abbreviations: TNM (tumor, node, metastasis); AST (aspartate amino transferase); AFP (alpha-fetoprotein); BMI (Body-Mass Index); METAVIR (meta-analysis of histological data in viral hepatitis); HCVAb (Hepatitis C-Virus antibody). **C.** Venn diagram plot for analysis of CMUH-HCC cohort for various assays, e.g., lipidome, transcriptome.


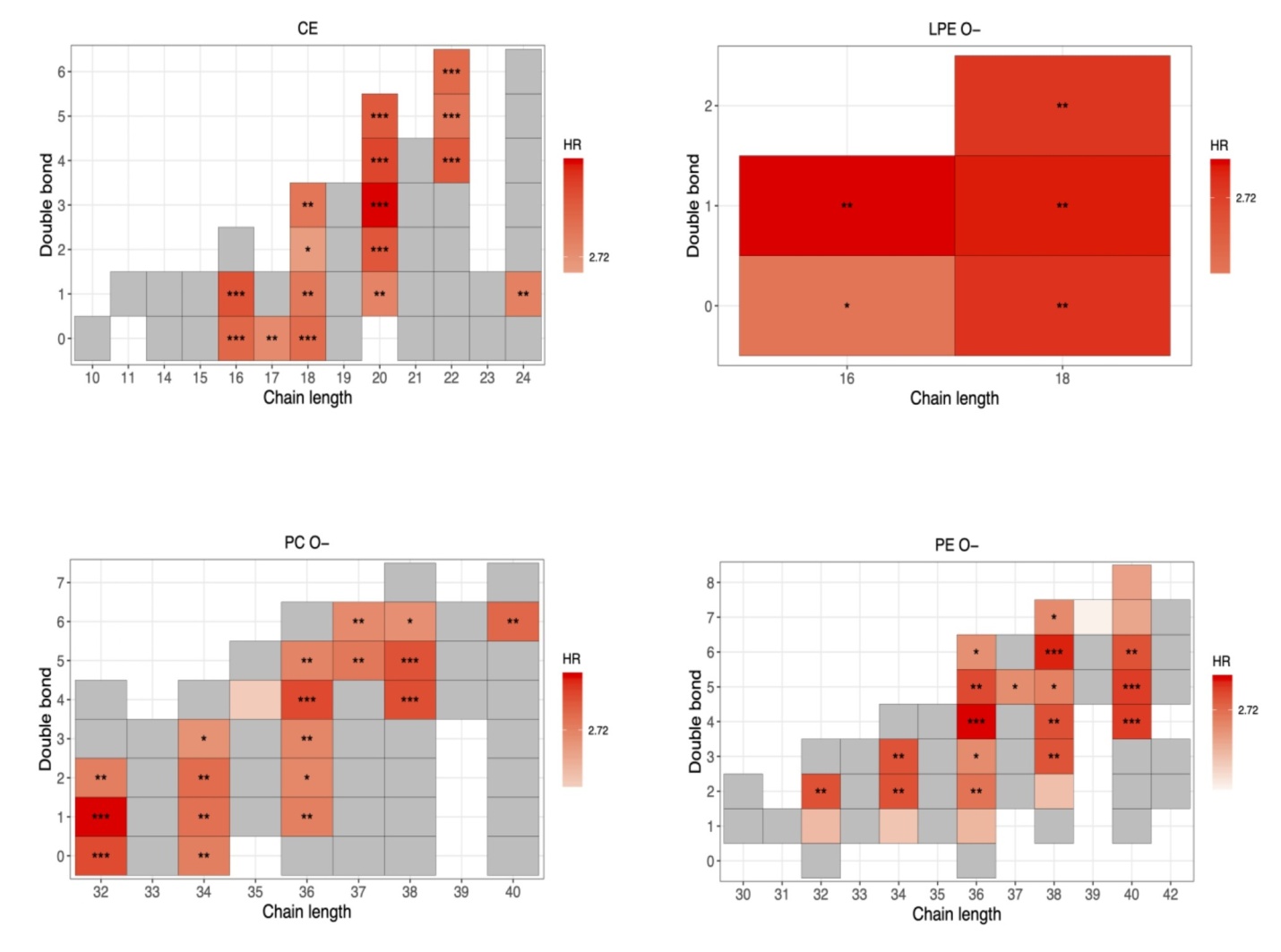


**Supplementary Figure S2. Analysis of chain length and double bonds in survival lipids.** This figure illustrates the relationship between the number of double bonds and the carbon chain length in the four lipid classes linked to hazard ratios (HR) in patients from the CMUH-HCC cohort.

**
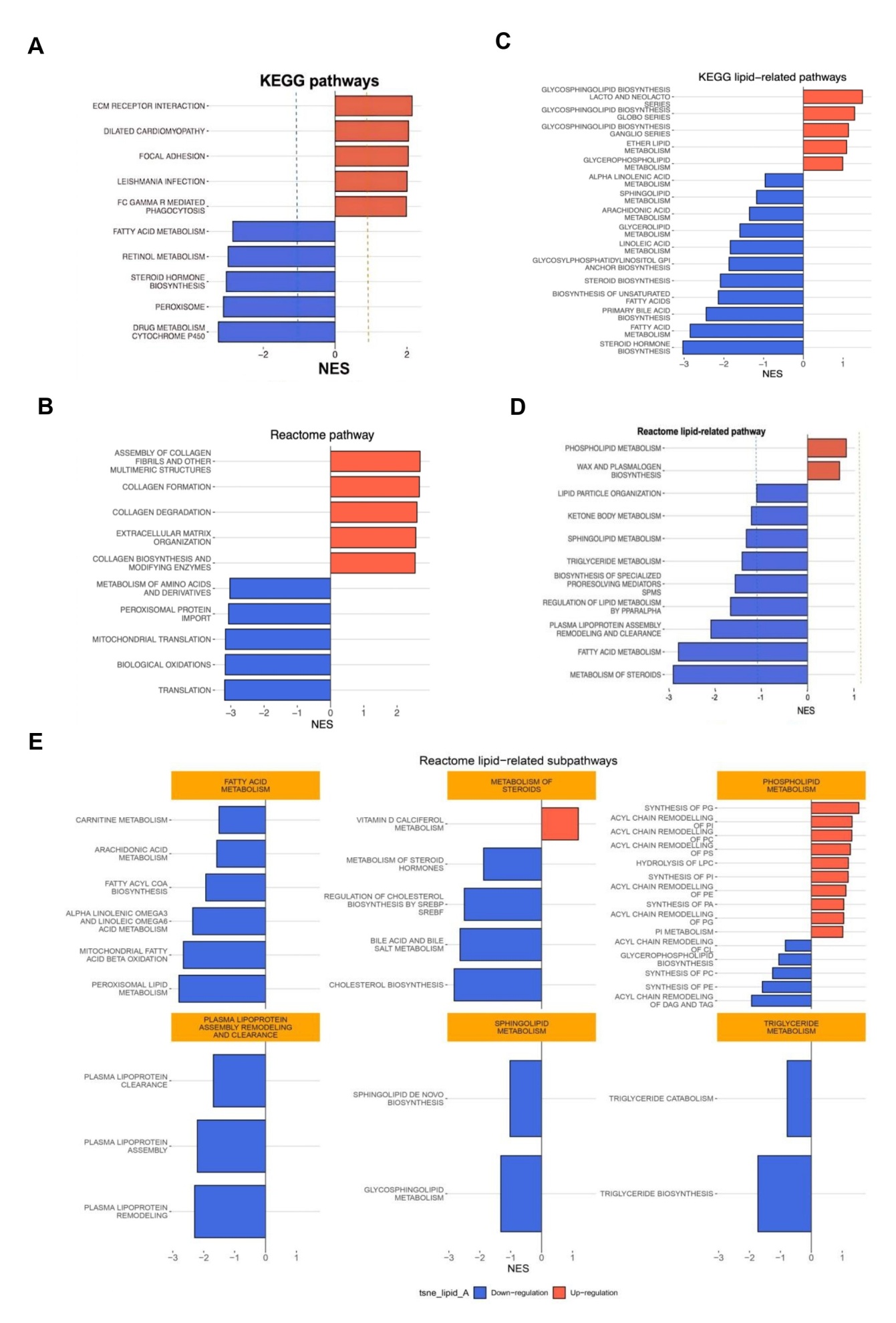
**

**Supplementary Figure S3. Functional enrichment analysis results of the significant genes of lipidomic tsne_A/B grouping. A.** All KEGG pathways. **B.** All Reactome pathway enrichment analysis. C. KEGG lipid-related pathway. **D.** Reactome lipid-related pathway. **E.** Reactome Lipid-related subpathway. NES: normalized enrichment score.

**
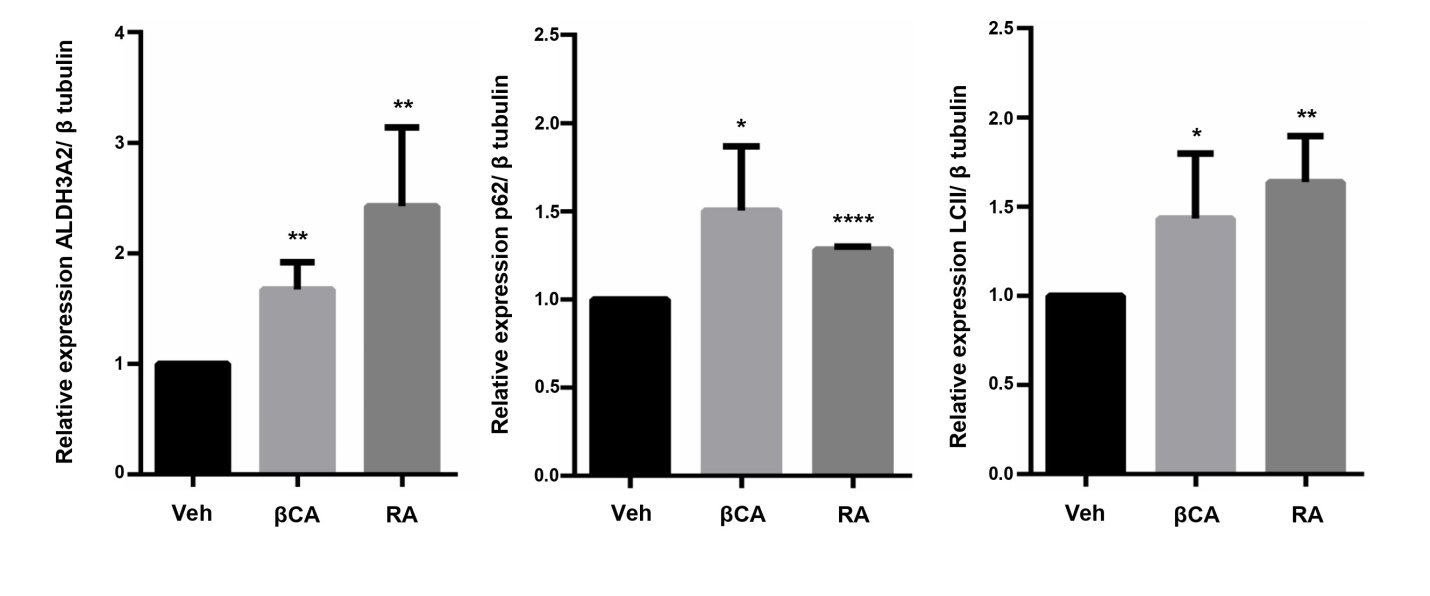
**

**Supplementary Figure S4. The quantification data of Figure 6A.** All in vitro results were derived from a minimum of three consistent experiments; * for p < 0.05, ** for p < 0.01, *** for p < 0.001, and **** for p < 0.0001.

**
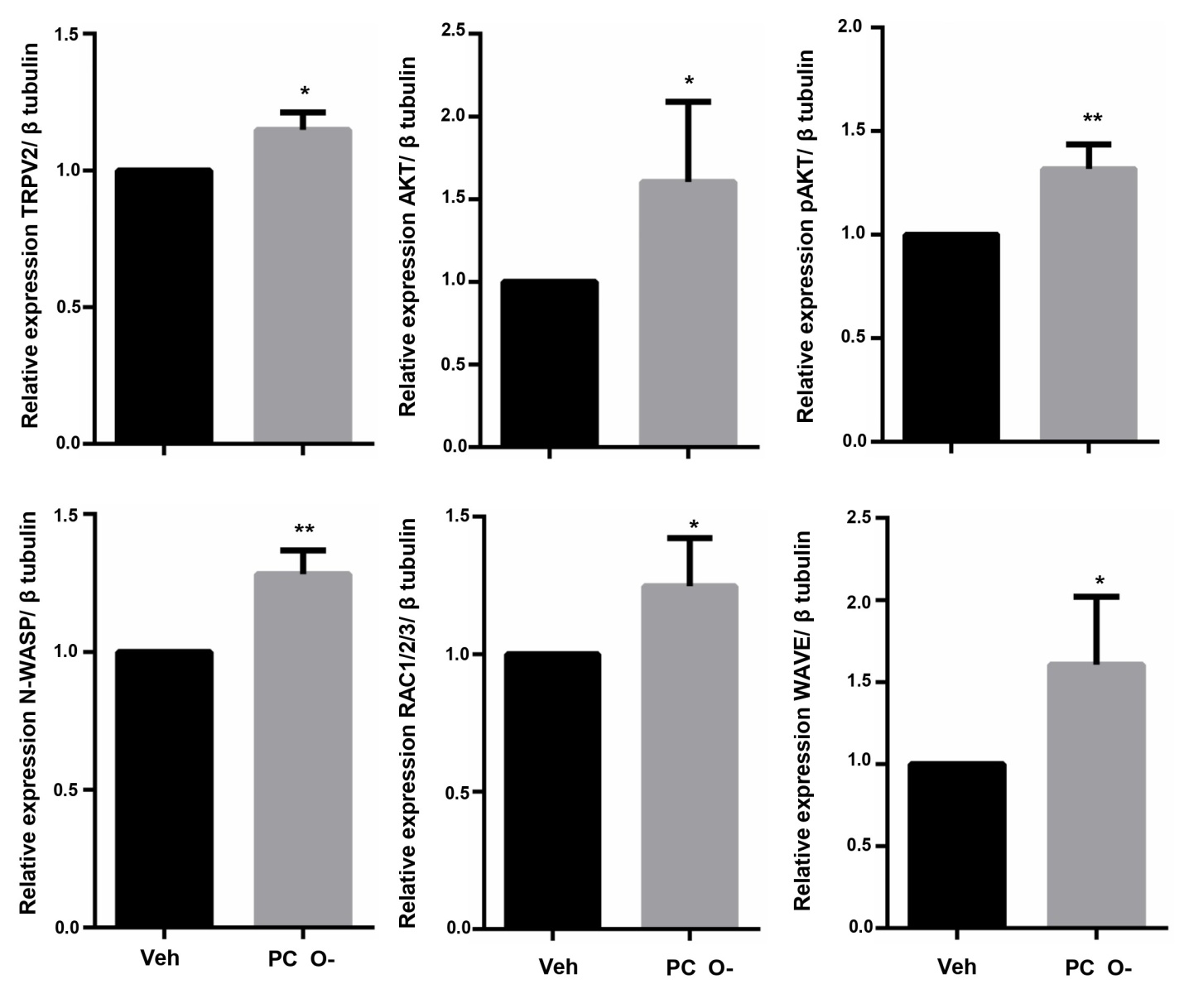
**

**Supplementary Figure S5. The quantification data of Figure 7A.** All in vitro results were derived from a minimum of three consistent experiments; * for p < 0.05, ** for p < 0.01.

**Table S1. Antibodies**

| Antibody | Application | Dilution | Cat no. | Company |
| --- | --- | --- | --- | --- |
| SQSTM1 | IF;IHC;WB | 1:100;1:200;1:1000 | ab91526 | Abcam |
| β-actin | WB | 1:1000 | C4;sc-47778 | Abcam |
| goat ant-rabbit IgG-HRP | WB | 1:5000 | sc-2004 | Santa Cruz |
| goat ant-mouse IgG-HRP | WB | 1:5000 | sc-2005 | Santa Cruz |
| β-tubulin | WB | 1:1000 | IR1-2 | iREAL |
| ALDH3A2 | IF;WB | 1:100; 1:500 | sc-373921 | Santa Cruz |
| LC3A/B | WB | 1:1000 | #12741 | Cell Signaling |
| Alexa-647 | IF | 1:200 | ab150075 | Abcam |
| ADRP | IF | 1:100 | sc-377429 | Santa Cruz |
| ADFP | IF | 1:100 | ab108323 | Abcam |
| LC3-B | IF | 1:100 | ab192890 | Abcam |
| LC3A/B | IF | 1:100 | Abc929 | Sigma-Aldrich |
| ALDH3A2 | IF | 1:100 | PA5-120435 | Invitrogen |
| N-WASP | WB | 1:1000 | #4848 | Cell Signaling |
| Rac1/2/3 | WB | 1:100 | sc-514583 | Santa Cruz |
| VRL-1 | WB | 1:100 | sc-514848 | Santa Cruz |
| WAVE | WB | 1:100 | sc-365165 | Santa Cruz |
| Akt | WB | 1:1000 | #4685 | Cell Signaling |
| Phospho-Akt(Ser473) | WB | 1:1000 | #4060 | Cell Signaling |

Abbreviations: IHC: immunohistochemical; WB: western blot; IF: immunofluorescence

**Table S2. Primers**

| Primer | Sequence | Application |
| --- | --- | --- |
| Human β-actin | F: TACCCCACACTGTGCCCATCTACGA  R: CAGCGGAACCGCTCATTGCCAATGG | Q-PCR |
| Human *PPARA* | F: CTATCATTTGCTGTGGAGATCG  R: AAGATATCGTCCGGGTGGTT | Q-PCR |
| Human *PPARB/D* | F: GTCACACAACGCTATCCGTTT  R: AGGCATTGTAGATGTGCTTGG | Q-PCR |
| Human *PPARG1* | F: CGTGGCCGCAGATTTGAA  R: CTTCCATTACGGAGAGATCCAC | Q-PCR |
| Human *PPARG2* | F: GGTGAAACTCTGGGAGATTCT  R: CTCTGTGTCAACCATGGTCA | Q-PCR |

**Table S3. Compound, lipids etc.**

| Name | Company | Cat no. |
| --- | --- | --- |
| 9-cis-Retinoic acid | Sigma-Aldrich | R4643 |
| β-Carotene | Sigma-Aldrich | C4582 |
| TRIzol | Invitrogen | 15596026 |
| Dulbecco’s Modified Eagle medium (DMEM) | GIBCO | 12100046 |
| Fetal bovine serum (FBS) | GIBCO | A3160601 |
| penicillin/streptomycin | CORNING | 30-002-CI |
| C16-18:1 PC | Avanti Research | 878112 |
| C16-18:1 PE | Avanti Research | 878130 |
| 1,2-Dioleoyl-sn-Glycero-3-Phosphocholine | Avanti Research | 850375P |
| DSPE-PEG(2000) Maleimide | Avanti Research | 880126P |
| D-alpha-tocopheryl polyethylene glycol 1000 succinate | Sigma-Aldrich | 57668 |
| phenol–chloroform pH 6.7/8.0 | VWR International | 0883-400 mL |
| Immobilon-P transfer membrane | Millipore | IPVH00010 |
| KAPA TM SYBR FAST One-Step qRT-PCR kit | KAPA biosystem | KM4100 |
| PrimeScript TM RT reagent kit | TAKARA Bio Inc | RR037A |
| 25 Culture-Inserts 2 Well for self-insertion | ibidi GmbH | IB-80209 |
| ECL reagent | Millipore | WBKLS0500 |
| ABC kit | Vector Laboratories, Inc. | NA |
| Dual-Luciferase®Reporter Assay System | Promega Corporation | E1960 |
| PPRE X3-TK-luc | Addgene | #1015 |
| Matrigel Basement Membrane Matrix Growth Factor | Corning Incorporated | 354230 |
| Millicell Hanging Cell Culture Inserts | Millipore | PTEP24H48 |
| four-well glass chamber slides | Millipore | PEZGS0416 |
| Dimethyl sulfoxide (DMSO) | Sigma-Aldrich | D8418 |
| Ethanol | Sigma-Aldrich | 51976 |
| BODIPY 493/503 | Thermo Fisher Scientific Inc. | D3922 |
| KAPATaq PCR Kit | KAPA biosystem | KK1014 |
| Triton X-100 | Bio Basic | TB0198 |
| DAPI | Sigma-Aldrich | D9542 |
| Mounting media | Invitrogen | P36930 |
| 96-well Clear Round Bottom Ultra-Low Attachment Microplate | Costar | 7007 |
| cytochalasin D | Enzo Biochem, Inc. | BML-T109 |
| Tranilast | Sigma-Aldrich | T0318 |
| Fenofibrate | Sigma-Aldrich | F6020 |
